# A sub-Riemannian model of the visual cortex with frequency and phase

**DOI:** 10.1186/s13408-020-00089-6

**Published:** 2020-07-29

**Authors:** E. Baspinar, A. Sarti, G. Citti

**Affiliations:** 1 MathNeuro Team, INRIA Sophia Antipolis, Valbonne, France; 2 EHESS, CAMS, Paris, France; 3 Department of Mathematics, University of Bologna, Bologna, Italy

**Keywords:** Sub-Riemannian geometry, Neurogeometry, Differential geometry, Gabor functions, Visual cortex, Image enhancement

## Abstract

In this paper, we present a novel model of the primary visual cortex (V1) based on orientation, frequency, and phase selective behavior of V1 simple cells. We start from the first-level mechanisms of visual perception, receptive profiles. The model interprets V1 as a fiber bundle over the two-dimensional retinal plane by introducing orientation, frequency, and phase as intrinsic variables. Each receptive profile on the fiber is mathematically interpreted as rotated, frequency modulated, and phase shifted Gabor function. We start from the Gabor function and show that it induces in a natural way the model geometry and the associated horizontal connectivity modeling of the neural connectivity patterns in V1. We provide an image enhancement algorithm employing the model framework. The algorithm is capable of exploiting not only orientation but also frequency and phase information existing intrinsically in a two-dimensional input image. We provide the experimental results corresponding to the enhancement algorithm.

## Introduction

The question of how we perceive has been an intriguing topic for different disciplines. One of the first schools that faced the problem is the Berlin school of experimental psychology, called *Gestalt* *psychology* school [68, 69, 103], which formulates precise laws explaining visual perception. The Gestalt psychology is a theory for understanding the principles underlying the emergence of perceptual units as the result of a grouping process. The main idea is that perception is a global phenomenon, which considers the scene as a whole and is much more than the pure sum of local perception. The first perceptual laws are of qualitative type, based on similarity, closure, good continuation, and alignment.

After that, there have been many psychophysical studies that attempted to provide a quantitative version of the grouping process. With the development of neuroscience studies, researchers started to look for cortical implementation of Gestalt laws, with a particular attention to neural architectures of the visual cortex. A particularly important one for our study is the pioneering work of Field et al. [43], which models Gestalt principles of good continuation and alignment. They experimentally proved that fragments aligned along a curvilinear path can be perceived as a unique perceptual unit much better than fragments with rapidly changing orientations. The results of their experiments were summarized in a representation, called *association fields*, which represent a complete set of paths with fixed initial position and orientation, which can be perceived as perceptual units.

The visual cortex is a part of the mammalian brain responsible for the first-level processing tasks of perceptual organization of local visual features in a visual stimulus (two-dimensional image). It is known from neurophysiological experiments that the visual cortex contains neurons (simple cells) that are locally sensitive to several visual features, namely, orientation [54–56, 58], spatial frequency [59–61, 77, 89, 90, 97, 98], phase [30, 72, 80, 86], scale [10], and ocular dominance [61, 71, 95].

The simple cells are organized in a hypercolumnar architecture, which was first discovered by Hubel and Wiesel [57]. In this architecture, a hypercolumn is assigned to each point $(x,y)$ of the retinal plane $M\simeq \mathbb {R}^{2}$ (if we disregard the isomorphic cortical mapping between retinal and cortical planes), and the hypercolumn contains all the simple cells sensitive to a particular value of the same feature type. The simple cells are able to locally detect features of the visual stimulus, and neural connectivity between the simple cells integrates them in a coherent global unity. Those two mechanisms, the feature detection and the neural connectivity, comprise the functional geometry of V1.

### Previous models and applications

Several models were proposed for the functional geometry of V1 associated with the simple cells that were only orientation sensitive. Early models date back to the 1980s. Koenderink and van Doorn [66, 67] revealed the similarity between Gaussian derivative functions and simple cell receptive profiles. They proposed visual models based on the functions of Gaussian derivatives as mathematical representations of the receptive profiles. Their findings indeed encouraged many studies relying on the choice of a family of Gaussian derivative functions and Gaussian kernels, among which we would like to mention the works of Young [104] and Lindeberg [74, 76].

A different modeling approach from the mentioned ones was employing Gabor functions as mathematical representations of the orientation-sensitive simple cell receptive profiles. The motivation for this choice was relying on an uncertainty principle as was elaborated by Daugman [29] through a generalization of the hypothesis of Marĉelja [78] (see also [62], where Jones and Palmer compared statistically the results obtained via Gabor functions and the neurophysiological results collected from V1 of a cat). Furthermore, Hoffman [51, 52] proposed to model the hypercolumnar architecture of V1 as a fiber bundle. Following the second school (which uses the Gabor functions) and further developing the model proposed by Petitot and Tondut [84] (see also Petitot [82, 83]), where hypercolumnar architecture was interpreted as a fiber bundle associated with a contact geometry, Citti and Sarti [25] introduced a group-based approach. They proposed a new model of the functional geometry of V1, which considered the sub-Riemannian geometry of the rototranslation group ($\mathrm {SE}(2)$) as a suitable model geometry. The main reason for employing $\mathrm {SE}(2)$ geometry was due to that the corresponding Lie algebra to $\mathrm {SE}(2)$ was providing a good model of the actual neural connectivity in V1. The model proposed in [25] has been extended to other visual features in addition to orientation, such as scale by Sarti et al. [92], and to other cell types such as complex cells sensitive to velocity and movement direction by Barbieri et al. [2] and Cocci et al. [27].

Furthermore, image processing applications employing Gabor transform to extract visual features from medical images were proposed by Duits et al. [38] (see also [94], where the phase of complex-valued outputs obtained from a multiscale orientation decomposition represented in a similarity group). In [38] an image enhancement together with an inpainting procedure was explained. Their method performs a multifrequency and multiphase Gabor transform on a two-dimensional image. The result of the transform is represented in the five-dimensional sub-Riemannian geometry of a reduced Heisenberg group. A combination of preprocessing via left-invariant diffusion and left-invariant convection–diffusion is applied for processing the lifted images to the Heisenberg group geometry. The convection provides sharpening of the image. Evolving phase of the diffused Gabor coefficients is handled via *phase-covariant* left-invariant convection–diffusion procedure by taking advantage of a Gabor transform. This Gabor transform takes into account spatial frequencies along both horizontal and vertical axes, differently from our case, where we consider spatial frequencies perpendicular to a single reference axis and provide a frequency decomposition along this axis for each orientation. Their procedure deals with the diffusion taking place in both phase and frequency dimensions, and it is applied to the detection of the cardiac wall deformations in MRI-tagging images. Our framework differs from this setting in two points. First of all, our geometry is derived from the receptive profile, Gabor function. The geometry is a natural result of the choice of the receptive profile. In other words, it is not assigned to the result of the Gabor transform as in [38]. Secondly, the model geometry we propose is not restricted to image processing. The image enhancement application we propose uses multifrequency channels. Its performance is comparable to multiscale methods and provides an improvement in comparison to a single-frequency method using a projected version of our model geometry. It uses pure Laplace–Beltrami evolution without any combination of pre/post-processing techniques or any additional nonlinear procedure. The motivation of the algorithm we propose is rather showing the importance of inclusion of frequency in cortical modeling and its potential to be an alternative framework to the multiscale image enhancement settings.

Other applications in medical image analysis employing scale and orientation information can be found in [18] and [63], where the Gabor transform is employed for the detection of local frequencies in tagging MRI (magnetic resonance imaging) images and thus for the computation of local frequency deformations in those images. The interested reader can also refer to [42] for different applications of geometric approach in computer vision and robotics. Additionally to those studies, the models in terms of cortical orientation and orientation-frequency selectivity, provided by Bressloff and Cowan [16, 17], can be useful references for the reader. We refer to [26] for a review of several cortical models including many mentioned ones.

We would like to address the reader to [14, 79], and [105] for some other both theoretical and practical aspects provided for sub-Riemannian image reconstruction/inpainting techniques. Moreover, such aspects are also provided and used in optimal control theory; see, for example, [13, 34]. The reader can refer to [19, 20] for image denoising approaches based on total-variation flows and to [21] for optimization approach used in image denoising and deblurring. Finally, we refer to [15] for a curvature-based approach, which can be applied to image denoising and inpainting.

### Neurophysiological motivation

The theoretical criterion underpinning the modeling we propose in this paper relies on the so-called neurogeometrical approach described by Citti and Sarti [25], Petitot and Tondut [84], and Sarti et al. [92]. Following this approach, processing capabilities of sensorial cortices and in particular of the visual cortex are modeled based on the geometrical structure of cortical neural connectivity. Global and local symmetries of the visual stimuli are captured by the cortical structure, which is invariant under those symmetries (see Sanguinetti et al. [91]). We follow a similar framework and will start from the first-level perceptual tasks performed by the simple cells from local feature extraction. This starting point will lead us to the model geometry of V1 associated with the simple cells sensitive to orientation, spatial frequency, and phase information at each position in a given two-dimensional image.

At the level of Gestalt organization, the neurogeometrical architecture in $\mathrm {SE}(2)$ [25] implements the psychophysical law of good continuation. The architecture in the affine group [92] implements good continuation and ladder (parallel chain of contours). The architecture in the Galilean group [2, 27] implements common fate. Finally, the architecture we consider here in a Gabor-based sub-Riemannian geometry implements similarity between textures/patterns and contains all the previous models employing the neurogeometrical approach.

It is known that the simple cells are selective not only to orientation but also to spatial frequency and the adjacent cells may have different phases. It was experimentally shown in [77] that the cortical architecture of a cat regarding spatial frequency of the stimulus is complementary to the hypercolumnar architecture associated with orientation. It was reported in [77] that the cortical neurons have the same preferred orientation but a variety of spatial frequency values along the electrode penetrations perpendicular to the cortical surface (i.e., along columns) and vice versa was valid for the penetrations parallel to the surface.

Deoxyglucose uptake increases in the regions of the brain where the neural activity increases. It was reported in [101] that the cats that were exposed to visual patterns containing a single spatial frequency and all orientations show columns of increased deoxyglucose uptake extending through all cortical layers. On the other hand, a stimulus containing all spatial frequencies and all orientations does not result in any difference in columnar density. Similar columnar organizations were reported in [96] and [12]. In [11], it was discovered that frequency preference maps were organized in frequency pinwheels around which all possible preferred frequency values are represented in an analogous way to the orientation pinwheels. In addition to those, it was shown in [61] that a wide range of frequency values were represented continuously in V1. Domains of different preferred frequency values were separated by $3/4$ mm (as in the case of hypercolumnar organization of orientation selectivity) at the extremes of the frequency continuum. Those frequency extremes were found mostly at the pinwheels. Finally, in [31], it was shown that the simple cells that are locally within the same cortical region respond to different frequencies. The range of the preferred frequency values of a cell does not overlap with the values of another nearby located one in the same cortical region. Those studies suggest a similar organization of frequencies to the orientation organization discovered by Hubel and Wiesel [55, 56].

Moreover, in [31] the simple cells were shown to be phase selective via a procedure based on the Enroth-Cugell–Robson *null phase test* [41] for spatial summation. The cell fires, does not respond, or performs inhibition on the grating pattern depending on the spatial position of the pattern with respect to the receptive field of the cell. Furthermore, Pollen and Ronner [87, 88] reported from their experiments that the adjacent simple cells in cat V1 that have a common preference for orientation and spatial frequency differ in spatial phase from each other by approximately $\pi /2$. This result is in coherence with that the receptive fields are conjugate pairs, that is, one even symmetric pair and one odd symmetric pair located around the same axis. Those experimental results support our choice of Gabor functions in such a way that adjacent simple cells can be interpreted as paired sine and cosine filters or Gabor functions. Finally, those aforementioned studies provide a neurophysiological basis for our choice of modeling the cortical architecture associated with frequency and phase selectivity in a similar columnar fashion as in the orientation selectivity case.

### Choice of receptive profile

Once the light reflects from a visual stimulus and arrives at the retina, it evokes some spikes, which are transmitted along the neural pathways to the simple cells in V1. Each simple cell gives a response called a *receptive profile* to those spikes. In other words, a receptive profile is the impulse response of a simple cell. The simple cells extract the information of local visual features by using their receptive profiles, and it is possible to represent the extracted features mathematically in a higher-dimensional space than in the given two-dimensional image plane. We call this space *the lifted space* or *the lifted geometry*. We will use an extended Gabor function as the receptive profile of the simple cells. We will see that this choice naturally induces the corresponding Lie algebra of the sub-Riemannian structure, which is the corresponding lifted geometry to our model. The Lie algebra and its integral curves model neural connectivity between the simple cells. Moreover, since some pairs of the algebra are not commutative, it is possible to formulate an uncertainty principle, and this principle is satisfied by the extended Gabor function. That is, the extended Gabor function minimizes uncertainties arising from simultaneous detection of frequency-phase and simultaneous detection of position-orientation (see also [33, Sect. 7.5], [1, 3, 4], and [94] for similar phenomena in different frameworks).

Concerning the question of which family of functions to use as receptive profiles, let us recall that receptive field models consisting of cascades of linear filters and static nonlinearities may be adequate to account for responses to simple stimuli such as gratings and random checkerboards, but their predictions of responses to complicated stimuli (such as natural scenes) are correct only approximately. A variety of mechanisms such as response normalization, gain controls, cross-orientation suppression, and intracortical modulation can intervene to change radically the shape of the profile. Then any static and linear model for the receptive profiles has to be considered just as a very first approximation of the complex behavior of a real dynamic receptive profile, which is not perfectly described by any of the static wavelet frames.

For example, the derivatives or differences of Gaussian functions are suitable approximations of the behavior of classical receptive profiles of the simple cells. Lindeberg [75, 76] starts from certain symmetry properties of the surrounding world and derives axiomatically the functions of Gaussian derivatives obtained from the extension of the family of rotationally symmetric Gaussian kernels to the family of affine Gaussian kernels, and proposes to model the simple cell receptive fields in terms of those Gaussian derivatives (see also Koenderink [66, 67], Young [104], and Landy and Movshon [70]). Indeed, Gaussian functions are good models of the receptive profiles if we restrict ourselves to the visual features except for frequency and phase. They provide good results for orientation and scale detection as shown by the scale-space school (see, e.g., the works of Lindeberg [73, 74, 76], Florack [44], ter Haar Romeny [99, 100], and Hannink et al. [50]). However, we are interested here in two-dimensional visual perception based on orientation, frequency, and phase-sensitive simple cells. Differently from the case with orientation-scale sensitive simple cells, frequency-phase sensitive simple cells cannot be modeled in a straightforward way by Gaussian derivative functions. A different order Gaussian derivative must be used for the extraction of each frequency component of a given image. This requires the use of different functions, each corresponding to a certain frequency and thus to a certain-order derivative. In other words, the frequency is not a parameter as in the case of scale, but each frequency corresponds to a different function. It is not possible to derive a natural geometry starting from the derivatives of the Gaussian, and it is rather required to assign an adequate geometric setting to the set of extracted feature values by the Gaussian derivatives to represent those values.

At this point, a Gabor function seems to be a good candidate for the detection of different orientation, frequency, and phase values in a two-dimensional image, since orientation, frequency, and phase are parameters of the Gabor function. In other words, instead of using different functions, we can use a single Gabor function corresponding to a set of parameter values to detect different feature values. In this way, we obtain a sub-Riemannian model geometry as the natural geometry induced directly by the Gabor function (i.e., by the receptive profile itself).

Moreover, the Gabor function is able to model both asymmetric simple cells and even/odd symmetric simple cells thanks to its phase offset term appearing in its wave content, whereas the functions of the Gaussian derivatives account only for the symmetric simple cells.

We take into account those aforementioned points and propose to use a Gabor function with frequency and phase parameters as the receptive profile model. The Gabor function allows us to extend the model provided in [25] to the true distribution of the profiles in V1 (including the asymmetric receptive profiles with phase shifts) in a straightforward way. Finally, we would like to refer to Duits and Franken [35–37], Franken and Duits [47], Sharma and Duits [94], Zhang et al. [106], and Bekkers et al. [9] for information about applications that employ other wavelets corresponding to unitary transforms for feature extraction.

Finally, the studies on group convolutional neural networks (G-CNN) are to be mentioned. A particularly relevant one to $\mathrm {SE}(2)$ sub-Riemannian geometry is explained by Bekkers [8]. Those neural networks use several neural layers for extraction and representation of the features necessary to perform proper high-level visual tasks such as object recognition. Feature extraction and the representation of the extracted features take advantage of the lifting of the image to a proper sub-Riemannian geometry (e.g., $\mathrm {SE}(2)$ geometry). Differently from the aforementioned approaches using a model function as a receptive profile, G-CNN *learns* the receptive profile through a feedback mechanism updating an initial arbitrary kernel by comparing the outputs of the whole network with the objects in the input image. The outputs are recognized objects by the neural network. We refer to [40] and [8] for a recent overview.

### Novelties

Here we consider the model framework provided in [25] as the departure point of our study. We extend this model from orientation selective framework to an orientation, frequency, and phase selective framework. Furthermore, we provide the neural connectivity among the simple cells not only orientation selective but also frequency selective with different phases. Thanks to the use of all frequency components of the Gabor functions, the Gabor transform can be followed by an exact inverse Gabor transform, which was not the case in the model presented in [25] since a single frequency component of the Gabor function was used. The projection of our generalized model onto $\mathrm {SE}(2)$ can be considered as equivalent to the model provided in [25]. The procedure we use to obtain the extended framework can be employed for the extension to a model associated with orientation-scale selective simple cells as well (see [7]).

We provide an image enhancement algorithm based on the Laplace–Beltrami procedure applied on all frequency channels in a reduced version of the model geometry. The reduced framework isolates each frequency and eliminates the activity propagation between different frequencies and phases. The Laplace–Beltrami procedure is then applied separately in each frequency channel and at a single phase, since in the reduced framework, each phase corresponds to a rotated version of the same image. It is an approximation of the Laplace–Beltrami procedure taking advantage of the full model geometry, and it partially employs the model geometry. In turn, it removes the excessive diffusion, which may destroy object boundaries and elongated structures in the image. Furthermore, it allows us to perform a three-dimensional Laplace–Beltrami procedure at every frequency instead of a five-dimensional Laplace–Beltrami in the full geometry, avoiding high computational and memory load, which is considerably heavy in the full five-dimensional model geometry. Finally, it provides the flexibility to process only the significant frequencies without altering the other frequency channels, which can be important in some texture images. This algorithm is inspired by the enhancement technique explained in [64], and it is different on three main points. The first point is that the technique in [64] relies on a Riemannian geometry and thus a Riemannian metric structure. We work in a sub-Riemannian geometry endowed with a sub-Riemannian metric. This results in that the sub-Riemannian differential operators are degenerate, that is, they perform in a subspace of the tangent space. The second point is that the technique presented in [64] employs a multiscale Gabor transform with fixed frequency and employs the scale as the additional feature to the orientation, whereas we employ a multifrequency Gabor transform with fixed scale and use the frequency as the additional feature for the enhancement. This results in that we can use exact the inverse Gabor transform when we project the processed lifted image in *G* to the two-dimensional image plane. The final point is the use of color. We study only grayscale images, but our approach has the potential to be extended to color images. Moreover, our sub-Riemannian model geometry is neurally inspired and induced by the receptive profiles. This is not the case in [64], where a proper Riemannian geometry is chosen for the image enhancement task, and it is not biologically motivated. Finally, we remark that the approach we use here for the construction of the model geometry is generic, and it provides a coherent way to derive the natural model geometry arising from the model function of the receptive profile.

### Outline

We will see in Sect. 2 the model structure. We will show how the model geometry with the associated horizontal connectivity can be derived starting from the receptive profile model, that is, from the Gabor function. Then in Sect. 3, we will provide explicit expressions of the horizontal integral curves with constant coefficients, which are considered as the models of the association fields in V1. Finally, in Sect. 4, we provide an image enhancement algorithm using the model framework together with the results obtained by applying a discrete version of the algorithm on some test images. We provide a conclusion in Sect. 5.

## The model

The model is based on two mechanisms. The first one is the linear feature extraction mechanism. The second mechanism is the horizontal connectivity, which models the neural connectivity in V1. We describe the model by using those two mechanisms in terms of both group and sub-Riemannian structures.

### Feature extraction and representation

#### Receptive profiles, symplectic structure, and contact form

Inspired by the receptive profile models proposed in [25] for the orientation selective behavior and in [2, 28, 32] for the spatio-temporal behavior of the simple cells, we propose to represent the receptive profile of a simple cell in our setting with the Gabor functions of the type

1 $$ \varPsi _{\alpha }(x,y,s):=\mathrm {e}^{-i (r \cdot (x-q_{1}, y-q_{2})-v(s- \phi ) )}\mathrm {e}^{-\lvert {x-q_{1}} \rvert ^{2}-\lvert {y-q_{2}} \rvert ^{2}} $$

with spatial frequency^1^$\omega >0$ and $r=(r_{1}, r_{2})=(-\omega \sin \theta, \omega \cos \theta )$, where we represent a point in a six-dimensional space $\mathcal{N}$ with $\alpha =(q_{1}, q_{2},\phi, r_{1}, r_{2}, v)\in \mathbb {R}^{6}$. The complex exponential is the wave content, and it is the main component detecting orientation, frequency, and phase of the objects in the given two-dimensional image. The second exponential is the Gaussian window. It provides the spatial localization around the point $(q_{1}, q_{2})$. The frequency *ω* determines how many wave peaks are found within the localizing window scaled by the Gaussian. The higher the number of wave peaks, the finer the structures that the Gabor can detect. The orientation *θ* determines how much the axis along which the waves lie deviate from the horizontal axis. It coincides with the orientation to which the simple cell associated with the Gabor function is sensitive. The parameter *ϕ* is the reference phase value, and it introduces a phase shift in the waves of the Gabor function as it varies. In the case of V1 motion sensitive cells with spatio-temporal dynamics, the term *v* represents the velocity of a two-dimensional plane wave propagation (see Barbieri et al. [2] for details). In our framework, we interpret $s-\phi $ as the phase centered at *ϕ* in a static sense. We are not interested in any temporal behavior or in motion sensitivity. Therefore we will fix *v* to a finite number.

Note that the coordinate variables are $(x,y,\theta,\omega, s)$. Here $(q_{1}, q_{2})$ and *ϕ* should be considered as fixed parameter values since they are the reference spatial position and phase values.

We may express *α* as a complex number $\alpha =(q_{1},q_{2},\phi )+i(r_{1},r_{2},v)\in \mathbb{C}^{3}$ and write the symplectic structure defined on the complex structure $\mathbb{C}^{3}$ (in which *α* is a fixed point) as follows [2, 45]:

$$ \varOmega =d\varTheta =\omega (\cos \theta\, dx+\sin \theta\, dy ) \wedge \,d \theta + (\sin \theta\, dx-\cos \theta \,dy )\wedge \,d\omega -dv \wedge \,ds. $$

The standard Liouville form follows from the symplectic structure by definition as

$$ \tilde{\varTheta }=r_{1}\,dx+r_{2}\,dy - v \,ds. $$

As was mentioned previously, we may fix *v* to a finite number in our static case. We choose $v=1$ for simplicity.

In this way, we obtain the five-dimensional space

2 $$ \mathcal{M}=\mathbb{R}^{2}\times S^{1}\times \mathbb {R}^{+}\times S^{1} \ni \alpha =\{q_{1}, q_{2},\theta,\omega,\phi \}=(q,z), $$

where *z* denotes the feature variables $(\theta, \omega ,\phi )\in S^{1}\times \mathbb {R}^{+}\times S^{1}$. Then we may write the associated Gabor function centered at $q\in M$ and sensitive to feature values *z* by using (1) as follows:

3 $$ \varPsi _{(q,z)}(x,y,s):=\mathrm {e}^{-i (\omega (-\sin \theta, \cos \theta ) \cdot (x-q_{1}, y-q_{2})-(s-\phi ) )}\mathrm {e}^{-\lvert {x-q_{1}} \rvert ^{2}-\lvert {y-q_{2}} \rvert ^{2}}. $$

The standard Liouville form $r_{1}\,dx+r_{2}\,dy - v \,ds$ reduces to

4 $$ \varTheta _{(\theta, \omega )}=r_{1} \,dx+r_{2} \,dy-ds=-\omega \sin \theta \,dx+ \omega \cos \theta\, dy-ds. $$

Indeed, *Θ* is a contact form since

5 $$ \varTheta \wedge d\varTheta \wedge d\varTheta = \omega \,dx \wedge \,dy \wedge d \theta \wedge \,d\omega \wedge \,ds $$

is a volume form. In other words, it is maximally nondegenerate and does not vanish at any point on the manifold $\mathcal{M}$.

The features which we would like to measure are embedded in the receptive profile. Those features manifest themselves as the plane waves corresponding to the Gabor functions in our model framework. Those plane waves describe the orthogonal directions to the simple cell connectivity. In other words, those plane waves are in orthogonal direction to the horizontal vector fields of the associated geometry, and their relation to the horizontal vector fields are found through the Liouville form. We may associate a Liouville form with each Gabor function given by (1). We write the Liouville form by following the coupling relations between the differential variables appearing in the wave content of the Gabor function. The considered differential variables in the Liouville form are not independent, but they are related to each other through a differential relation. The vanishing of the Liouville form expresses this differential constraint, and hence it reduces the dimension of free variables on the tangent space. This defines the horizontal tangent space and thus the horizontal vector fields.

#### Set of receptive profiles

An important property of Gabor functions is that they are invariant under certain symmetries. Therefore any Gabor function can be obtained from a reference Gabor function (mother Gabor function) up to a certain transformation law.

Let us denote the origin for the layer of a frequency *ω* by $0_{\omega }=(0,0,\omega,0)\in \mathcal{M}$. Then a suitable choice of the mother Gabor function with frequency *ω* is

6 $$ \varPsi _{0_{\omega }}(x,y,s)=\mathrm {e}^{-i(\omega y-s )}\mathrm {e}^{-x^{2}-y^{2}}. $$

We set

7 $$ A_{(q,\theta,\phi )}(\tilde{x},\tilde{y},\tilde{s})= \begin{pmatrix} q_{1} \\ q_{2} \\ \phi \end{pmatrix} + \begin{pmatrix} \cos \theta & -\sin \theta & 0 \\ \sin \theta & \cos \theta & 0 \\ 0 & 0 & 1 \end{pmatrix} \begin{pmatrix} \tilde{x} \\ \tilde{y} \\ \tilde{s} \end{pmatrix} =(x,y,s), $$

which describes at each frequency the relation between a generic receptive profile centered at $z=(q,\theta,\omega,\phi )$ and the mother Gabor function through

8 $$ \varPsi _{(q,z)}(x,y,s)=\varPsi _{0_{\omega }} \bigl(A^{-1}_{(q,\theta,\phi )}(x,y,s) \bigr). $$

The set of all receptive profiles obtained from the mother Gabor function with all possible combinations of feature values at each possible frequency is called the *set of receptive profiles*.

#### Output of a simple cell

We obtain the output response of a simple cell (which is located at the point $q=(q_{1},q_{2})\in M\simeq \mathbb {R}^{2}$ and sensitive to the feature values $z=(\theta, \phi, \omega )$) to a generic image $I: M\rightarrow \mathbb {R}$ as a convolution with Gabor filter banks:

9 $$ O^{I}(q,z)= \int _{M} I(x,y)\varPsi _{(q,z)}(x,y,0) \,dx\, dy. $$

We apply the convolution for all feature values *z* at every point *q* to obtain the output responses of all receptive profiles in the set of receptive profiles. It is equivalent to applying a multifrequency Gabor transform on the given two-dimensional image. Since we use all frequency components of the transform, we can employ the exact inverse Gabor transform to obtain the initial image:

10 $$ I(q)= \int _{\mathcal{M}} O^{I}(x,y, z)\bar{\varPsi }_{(x,y,z)}(q,0) \,dx\, dy \,dz $$

with *Ψ̄* denoting the complex conjugate. We will call the output response *lifted image* and the Gabor transform *lifting*.

Note that the theory provided in [38] takes into account the complex-valued functions resulting from the Gabor transform and explains a proper way to apply image enhancement and inpainting on the complex structure. Differently from this example, cortical models have employed the real part, imaginary part, or the absolute value of the output responses resulting from the convolution with corresponding Gabor filters so far (see, e.g., [25, 92, 93]). In other words, they have not taken into account half of the information they obtained from an image. Furthermore, the inverse Gabor transform was not possible in the previous models of the visual cortex given in [25, 92, 93] since in those models a single-frequency Gabor transform was employed to obtain the output responses.

We remark that we consider the whole complex structure of the result of the convolution (9) as the output response of a simple cell and use the exact inverse Gabor transform (10) for the reconstruction of the image. This formula is adapted from [38, Sect. 2] as explained in [5, Chap. 8] (see also [36] for explanations of wavelet reconstruction formulas, in particular, in the case of so-called *cake wavelets*).

### Horizontal vector fields and connectivity

Horizontal vector fields are defined as the elements of

11 $$ \operatorname{ker}\varTheta =\bigl\{ X \in T\mathcal{M}: \varTheta (X)=0\bigr\} , $$

where $T\mathcal{M}$ denotes the tangent bundle of the five-dimensional manifold $\mathcal{M}$. They are naturally induced by the 1-form *Θ* given in (4). The horizontal vector fields are found explicitly as

12 $$\begin{aligned} \begin{aligned}& X_{1} =\cos \theta \partial _{x}+\sin \theta \partial _{y},\qquad X_{2} =\partial _{\theta }, \\ &X_{3} =-\sin \theta \partial _{x}+\cos \theta \partial _{y}+ \omega \partial _{s},\qquad X_{4} =\partial _{\omega }. \end{aligned} \end{aligned}$$

The corresponding horizontal distribution is therefore as follows:

13 $$ \mathcal{D}^{\mathcal{M}}=\operatorname{span}(X_{1},X_{2},X_{3},X_{4}). $$

All nonzero commutators related to the horizontal vector fields given in (12) are as follows:

14 $$\begin{aligned} \begin{aligned}&[X_{1}, X_{2}]= \sin \theta \partial _{x}-\cos \theta \partial _{y}, \\ &[X_{2}, X_{3}]= -\cos \theta \partial _{x}-\sin \theta \partial _{y}, \\ &[X_{3}, X_{4}]= -\partial _{s}. \end{aligned} \end{aligned}$$

Note that the horizontal vector fields are bracket generating since

15 $$ T_{\alpha }\mathcal{M}=\operatorname{span} \bigl(X_{1}, X_{2}, X_{3}, X_{4}, [X_{1}, X_{2}]\bigr) (\alpha ) $$

for all $\alpha \in \mathcal{M}$, where $T_{\alpha }\mathcal{M}$ denotes the tangent space of $\mathcal{M}$ at *α*. Obviously, (15) shows that the horizontal vector fields fulfill the Hörmander condition [53], and consequently they provide the connectivity of any two points on $\mathcal{M}$ through the horizontal integral curves defined on $\mathcal{M}$ due to the Chow–Rashevski theorem [23]. This connectivity property is particularly important since it guarantees that any two points in V1 can be connected via the horizontal integral curves, which are the models of the neural connectivity patterns in V1.

### Functional architecture of the visual cortex

#### The architecture as a Lie group

Receptive profiles evoke a group structure at each frequency $\omega \in \mathbb {R}^{+}$. We can describe the group structure underlying the set of receptive profiles by using the transformation law given in (7).

First, we notice that the elements $(q,\theta,\phi )$ induce the group given by

16 $$ G_{\omega }\simeq \bigl\{ A_{(q,\theta,\phi )}: (q,\theta,\phi )\in M \times S^{1}\times S^{1}\bigr\} , $$

which is indeed a Lie group associated with fixed frequency *ω*.

Then using (7), we write the group multiplication law for two elements

17 $$ g=\bigl(q^{g},\theta _{1},\phi _{1}\bigr),\qquad h= \bigl(q^{h}, \theta _{2},\phi _{2}\bigr),\quad g,h\in G_{\omega }, $$

as

18 $$ g h= \left( \begin{pmatrix} q^{g}_{1} \\ q^{g}_{2} \end{pmatrix} +R_{\theta _{1}+\theta _{2}} \begin{pmatrix} q^{h}_{1} \\ q^{h}_{2} \end{pmatrix} , \theta _{1}+\theta _{2}, \phi _{1}+\phi _{2} \right). $$

The differential $L_{g^{\ast }}$ of the left-translation

19 $$\begin{aligned} \begin{aligned}& L_{g}: G_{\omega } \rightarrow G_{\omega }, \\ &\phantom{L_{g} : }h \mapsto g h \end{aligned} \end{aligned}$$

is given by

20 $$ L_{g^{\ast }}= \begin{pmatrix} \cos (\theta ) & 0 & -\sin (\theta ) & 0 \\ \sin (\theta ) & 0 & \cos (\theta ) & 0 \\ 0 & 1 & 0 & 0 \\ 0 & 0 & \omega & 0 \end{pmatrix} . $$

The vector fields $X_{1}$, $X_{2}$, and $X_{3}$ are bracket generating due to that

21 $$ \operatorname{span}\bigl(X_{1}, X_{2}, X_{3}, [X_{1}, X_{2}] \bigr) (g)=T_{g}G_{ \omega } $$

for every $g\in G_{\omega }$. Hence $X_{1}$, $X_{2}$, and $X_{3}$ generate the Lie algebra corresponding to $G_{\omega }$.

#### The architecture as a sub-Riemannian structure

The functional geometry is associated with a sub-Riemannian structure at each frequency *ω*. We denote by $G_{\omega }$ the submanifold of $\mathcal{M}$ with points $h=(q,\theta,\phi, \omega )=(q,z)$ restricted to a fixed *ω*. In this case the horizontal distribution is found by

22 $$ \mathcal{D}^{G_{\omega }}=\operatorname{span}( X_{1}, X_{2}, X_{3} ). $$

Furthermore the induced metric $(g_{ij})^{G_{\omega }}_{h}: \mathcal{D}^{G_{\omega }}\times \mathcal{D}^{G_{ \omega }}\rightarrow \mathbb {R}$ is defined on $\mathcal{D}^{G_{\omega }}$ and at every point $h\in G_{\omega }$ makes $X_{1}, X_{2}, X_{3} $ orthonormal.

Finally, the associated sub-Riemannian structure with frequency *ω* is written as the triple

23 $$ \bigl(G_{\omega }, \mathcal{D}^{G_{\omega }}, (g_{ij})_{h}^{G_{\omega }} \bigr). $$

## Horizontal integral curves

The lifting mechanism leaves each lifted point isolated from each other since there is no connection between the lifted points. Horizontal vector fields endow the model with an integration mechanism that provides an integrated form of the local feature vectors obtained from the lifted image at each point on $\mathcal{M}$.

Once a simple cell is stimulated, its activation propagates between the simple cells along certain patterns, which can be considered as the integrated forms of the local feature vectors. This propagation machinery is closely related to the association fields [43], which are the neural connectivity patterns between the simple cells residing in different hypercolumns (long-range horizontal connections) within V1. The association fields coincide with the anisotropic layout of the long-range horizontal connections at the psychophysical level. In the classical framework of [25], those association fields were modeled as the horizontal integral curves of $\mathrm {SE}(2)$. We follow a similar approach and propose to model the association fields in our model framework as the horizontal integral curves associated with the five-dimensional sub-Riemannian geometry of $\mathcal{M}$. We conjecture that those horizontal integral curves coincide with the long-range horizontal connections between orientation, frequency, and phase selective simple cells in V1.

We denote a time interval by $\mathcal{I}=[0,T]$ with $0< T<\infty $ and then consider a horizontal integral curve $(q_{1},q_{2},\theta, \omega ,\phi )=\gamma:\mathcal{I}\rightarrow \mathcal{M}$ associated with the horizontal vector fields given in (12) and starting from an initial point $\hat{\alpha }=(\hat{q}_{1},\hat{q}_{2},\hat{\theta },\hat{\omega }, \hat{\phi })$. Let us denote the velocity of *γ* by $\gamma ^{\prime }$. At each time $t\in \mathcal{I}$ the velocity is a vector $\gamma ^{\prime }(t)\in \operatorname{span}(X_{1},X_{2},X_{3},X_{4}) (\gamma (t) )$ at $\gamma (t)=(q_{1}(t),q_{2}(t),\theta (t),\omega (t),\phi (t))\in \mathcal{M}$. To compute the horizontal integral curves, we first consider the vector field $\gamma ^{\prime }$ given by

24 $$\begin{aligned} \gamma ^{\prime }(t)=X\bigl(\gamma (t)\bigr)= (c_{1} X_{1}+ c_{2} X_{2}+c_{3} X_{3}+c_{4} X_{4}) \bigl(\gamma (t)\bigr),\quad t\in \mathcal{I}, \end{aligned}$$

with coefficients $c_{i}$ (which are not necessarily constants), $i\in \{1,2,3,4 \}$. Then we can write each component of $\gamma ^{\prime }(t)$ as follows:

25 $$\begin{aligned} \begin{aligned} &q^{\prime }_{1}(t) =c_{1}\cos \bigl(\theta (t)\bigr)-c_{3}\sin \bigl(\theta (t) \bigr), \\ &q^{\prime }_{2}(t) =c_{1}\sin \bigl(\theta (t) \bigr)+c_{3}\cos \bigl(\theta (t)\bigr), \\ &\theta ^{\prime }(t) = c_{2}, \\ &\omega ^{\prime } (t) = c_{4}, \\ &\phi ^{\prime }(t) = c_{3} \omega (t). \end{aligned} \end{aligned}$$

In the case where the coefficients $c_{i}$ are real constants and $c_{2}\neq 0$, we solve the system of ordinary differential equations (25) of *t* with initial condition *α̂* and find the following solution:

26 $$\begin{aligned} \begin{aligned}& q_{1}(t) = \hat{q}_{1}+\frac{1}{c_{2}} \bigl(-c_{3}\cos (\hat{\theta })+c_{3} \cos (c_{2} t+\hat{\theta })-c_{1}\sin ( \hat{\theta })+c_{1}\sin (c_{2} t+ \hat{\theta }) \bigr), \\ &q_{2}(t) =\hat{q}_{2}+\frac{1}{c_{2}} \bigl(c_{1}\cos (\hat{\theta })-c_{1} \cos (c_{2} t+ \hat{\theta })-c_{3}\sin (\hat{\theta })+c_{3}\sin (c_{2} t+ \hat{\theta }) \bigr), \\ &\theta (t) = c_{2} t+\hat{\theta }, \\ &\omega (t) = c_{4} t+\hat{\omega }, \\ &\phi (t) = \frac{1}{2} \bigl(c_{3} c_{4} t^{2}+2 t c_{3} \hat{\omega }+2 \hat{\phi } \bigr). \end{aligned} \end{aligned}$$

If $c_{2}=0$, then the solution becomes

27 $$\begin{aligned} \begin{aligned} q_{1}(t) & = \hat{q}_{1}+t \bigl(c_{1}\cos (\hat{\theta })-c_{3} \sin ( \hat{\theta }) \bigr), \\ q_{2}(t) & =\hat{q}_{2}+t \bigl( c_{3}\cos (\hat{ \theta })+c_{1}\sin ( \hat{\theta }) \bigr), \\ \theta (t) & =\hat{\theta }, \\ \omega (t) & =c_{4} t+\hat{\omega }, \\ \phi (t) & =\frac{1}{2}\bigl(c_{3}c_{4} t^{2}+2 t c_{3} \hat{\omega }+2 \hat{\phi }\bigr). \end{aligned} \end{aligned}$$

Note that (26) and (27) describe a family of horizontal integral curves described by the horizontal distribution

$$ \mathcal{D}^{\mathcal{M}}= \bigcup_{\omega \in \mathbb {R}^{+}} \mathcal{D}^{G_{\omega }}=\operatorname{span}( X_{1}, X_{2}, X_{3}, X_{4}). $$

We are interested rather in two specific subfamilies corresponding to the horizontal vector fields that reside in one of the two orthogonal $\mathcal{D}_{\alpha }^{\mathcal{M}}$ subspaces defined at every point $\alpha =(q,\theta, \omega ,\phi )\in \mathcal{M}$ as

28 $$ S_{1}\mathcal{D}^{\mathcal{M}}_{\alpha }=\operatorname{span}(X_{1},X_{2}) ( \alpha ),\qquad S_{2}\mathcal{D}^{\mathcal{M}}_{\alpha }= \operatorname{span}(X_{3}, X_{4}) (\alpha ), $$

satisfying

29 $$ \mathcal{D}^{\mathcal{M}}_{\alpha }=S_{1}\mathcal{D}^{\mathcal{M}}_{\alpha } \oplus S_{2}\mathcal{D}^{\mathcal{M}}_{\alpha }. $$

**Figure 1:**
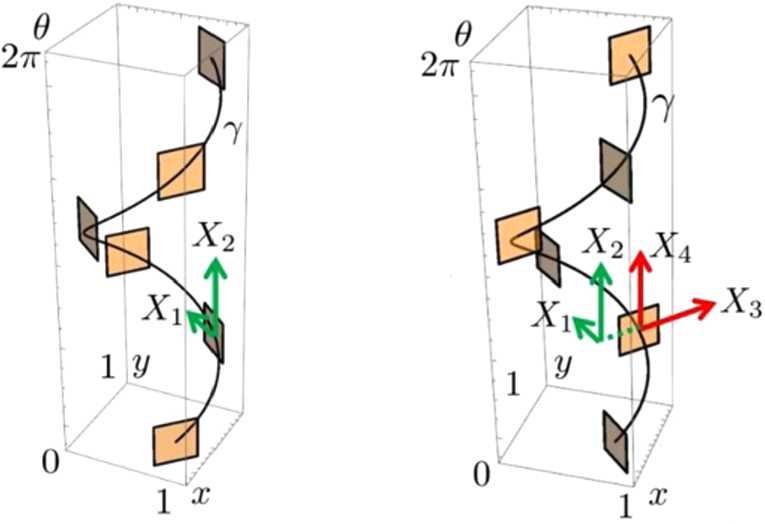
An integral curve along the vector field $X_{1}+X_{2}$. It represents an orientation fiber once *ω* and *ϕ* are fixed. The tangent planes spanned by $X_{1}$, $X_{2}$ (left) and $X_{3}$, $X_{4}$ (right) are shown at six points on the curve

**Figure 2:**
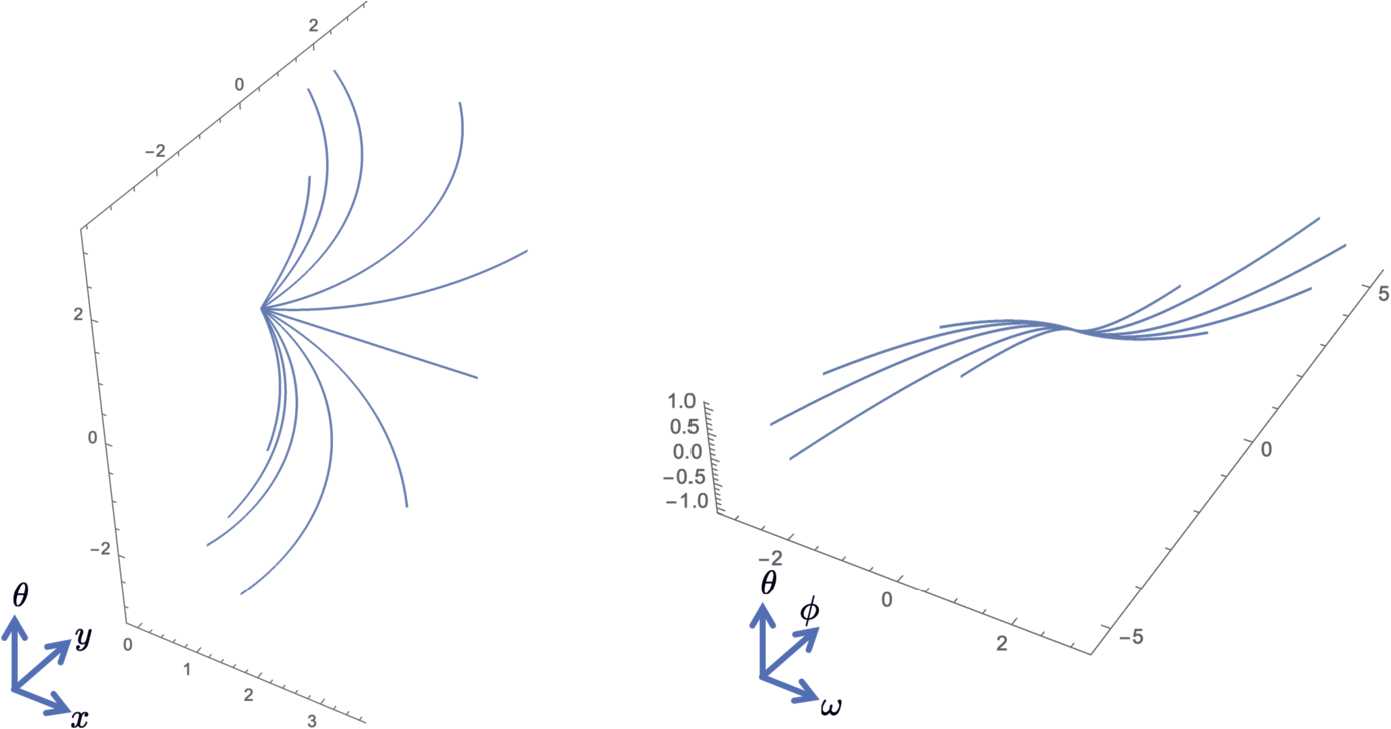
Integral curve fans corresponding to $X_{1}+c_{2} X_{2}$ (left) and $X_{3}+c_{4} X_{4}$ (right) where $c_{2}$ and $c_{4}$ are varied, respectively

Figure 1 gives an illustration of the orthogonal layout of $S_{1}\mathcal{D}^{\mathcal{M}}_{\alpha }$ and $S_{2}\mathcal{D}^{\mathcal{M}}_{\alpha }$ at points *α* on an orientation fiber, that is, on a horizontal integral curve along $X_{1}+X_{2}$ corresponding to some fixed *ω* and *ϕ*. See also Fig. 2, where the integral curves along the vector fields $X_{1}+c_{2} X_{2}$ and $X_{3}+c_{4} X_{4}$ with varied $c_{2}$ and $c_{4}$ values, respectively, are presented.

We remark that $S_{1}\mathcal{D}^{\mathcal{M}}_{\alpha }$ is the horizontal tangent space $T_{(q,\theta )}\mathrm {SE}(2)$ of $\mathrm {SE}(2)$ at the point *α* once the frequency *ω* and phase *ϕ* are fixed. In other words, at each point $\alpha =(q,\theta, \omega ,\phi )$ with *ω* and *ϕ* fixed on $\mathcal{M}$, we find the submanifold $\mathrm {SE}(2)$, which is the classical sub-Riemannian geometry corresponding to the model given in [25]. This property allows the simple cell activity to be propagated in each subspace corresponding to a frequency-phase pair separately, and it will be important for image enhancement applications employing our model framework.

Finally, we note that the notion of horizontal curves is mathematically more general than that we consider in our model framework. It refers to the integral curves that are everywhere tangent to the horizontal tangent space. Therefore the coefficients may be time varying as well.

## Enhancement

Image enhancement refers to smoothing a given input image by reducing the noise and at the same time preserving the geometric structures (edges, corners, textures, etc.). We perform our image enhancement procedure on the output responses instead of those on the input image. Since the output responses encode the local feature values of orientation, frequency, and phase, this allows us to exploit the additional information obtained from those features. Our enhancement procedure is based on an iterative Laplace–Beltrami procedure on the simple cell output responses in the five-dimensional sub-Riemannian geometry $\mathcal{M}$, and it results in a mean curvature flow in the geometry.

### Laplace–Beltrami procedure

An anisotropic metric on the space $\mathcal{M}$ of simple cell output responses defines the sub-Riemannian Laplacian in the sub-Riemannian space generated by the simple cells:

30 $$ \Delta _{0} u=\sum_{i=1}^{4} c_{i} X_{i}X_{i} u, $$

where the coefficients $c_{i}$ are nonnegative constants representing the weights of the second-order horizontal vector fields given in (12). The weights are used to adjust the operator to the sub-Riemannian homogeneity of $\mathcal{M}$. They are particularly important in the discrete case, where different dimensions of the space need not necessarily be sampled in the same way.

It has been proved by Franceschiello et al. [46] that the output induces a metric on the space of the model geometry proposed in [25] and the metric elicits certain visual illusions. Franceschiello et al. [46] used a simplified diagonal metric. On the other hand, following the approach of Kimmel et al. [64, 65], we choose the metric induced by the output $O^{I}(q, z)$ on $\mathcal{M}$ and use a simplified version of this metric for applications.

The metric $(g_{ij})$ induced by the output responses is defined as follows.

#### Definition 1

31 $$ (g_{ij})= \begin{pmatrix} 1+c_{1}(X_{1}u)^{2} & \sqrt{c_{1}c_{2}}X_{1}uX_{2} u & \sqrt{c_{1}c_{3}}X_{1}uX_{3} u & \sqrt{c_{1}c_{4}}X_{1}uX_{4} u \\ \sqrt{c_{1}c_{2}} X_{2}u X_{1} u & 1+ c_{2} (X_{2}u)^{2} & \sqrt{c_{2}c_{3}} X_{2}uX_{3} u & \sqrt{c_{2}c_{4}} X_{2}uX_{4} u \\ \sqrt{c_{1}c_{3}} X_{3}u X_{1} u & \sqrt{c_{2}c_{3}} X_{3}uX_{2} u & 1+ c_{3} (X_{3} u)^{2} & \sqrt{c_{3}c_{4}} X_{3}uX_{4} u \\ \sqrt{c_{1}c_{4}} X_{4}uX_{1} u & \sqrt{c_{2}c_{4}} X_{4}uX_{2} u & \sqrt{c_{3}c_{4}}X_{4}uX_{3} u & 1+c_{4} (X_{4} u)^{2} \end{pmatrix} $$

with constants $c_{1},c_{2},c_{3},c_{4}\geq 0$.

We denote the inverse metric by $(g^{ij})$ and its elements by $g^{ij}$.

The mean curvature flow provides an adapted enhancement to the surface underlying the image function *I* since the flow is restricted to the evolving level sets of the image. The Laplace–Beltrami procedure is a generalization of Laplacian from flat spaces to manifolds. It restricts the diffusion of a function to the manifold on which the function is defined thanks to the metric given in Definition 1. Metrics of type (1) are commonly used on Riemannian manifolds where the induced metric is found in terms of the vector fields spanning the whole tangent space at each point on the manifold. In the sub-Riemannian setting in our model, the metric is composed by the horizontal vector fields. Those vector fields span the horizontal subset of the whole tangent space at each point. Therefore the model geometry is degenerate. Moreover, the horizontal vector fields are noncommutative, and this results in some diffusion taking place in orthogonal directions to the manifold. Such a diffusion should be kept small; otherwise, it might result in excessive blurring, which destroys contextual information (contours, object boundaries, etc.) in the image. This is one of the reasons for us to use a reduced version of the metric given in Definition 1. Yet, the noncommutative nature of the vector fields provides the full connectivity of the geometry through the horizontal integral curves due to the Hörmander condition and Chow–Rashevski theorem.

The coefficients $c_{1}, c_{2}, c_{3}, c_{4}$ can be chosen suitably to determine the amount of diffusion in the orthogonal direction. The Laplace–Beltrami operator is written as

32 $$ L u=\sum_{i,j=1}^{4} \frac{1}{\sqrt{\operatorname{det}(g_{ij})}}X_{i} \bigl(\sqrt{ \operatorname{det}(g_{ij})}g^{ij}X_{j} u \bigr), $$

where $\operatorname{det}(g_{ij})$ is the determinant of the induced metric. The Laplace–Beltrami operator can be considered as the linearization of the motion by curvature explained in [6]. It performs a more adaptive (and restricted) diffusion to the surface in comparison to the horizontal diffusion characterized by (30).

For practical reasons, we will use the Laplace–Beltrami process with operator (32) associated with a reduced version of the metric provided in Definition 1. It is equivalent to consider each frequency channel as a separate space. It provides the freedom to work on a Laplace–Beltrami procedure separately in each frequency subspace differently from a nonlinear diffusion in a single-frequency channel. We refer to [24] for an example of a single-frequency Laplace–Beltrami procedure restricted to the sub-Riemannian metric of $\mathrm {SE}(2)$ and its application to image inpainting and enhancement. In addition to a Laplace–Beltrami procedure, other procedures based on total-variation flows in sub-Riemannian geometries for successful image processing can be found in [22, 24]. Finally, we address the reader to [39] for some generalizations of total-variation and mean curvature flows to $\text{SE}(d)\simeq \mathbb {R}^{3}\rtimes S^{d-1}$ and their applications in the higher-dimensional sub-Riemannian space of $\text{SE}(3)$.

The evolution equation for the enhancement via a sub-Riemannian Laplace–Beltrami procedure is written as

33 $$ \textstyle\begin{cases} \partial _{t} u=L u, \\ u_{|t=0}=O^{I}(q, p), \end{cases} $$

for all $(q,p)\in \mathcal{M}$ and $0< t\leq T$. Here the operator *L* depends on the metric evolving at each time instant due to the evolving output responses. This evolution equation models the neural activity propagation between simple cells along the neural connections modeled via the horizontal connectivity in our framework.

#### Reduced equation

It is possible to perform the Laplace–Beltrami procedure in each frequency and phase subspace separately in a reduced framework. In that case, we choose $c_{1},c_{2}> 0$ and $c_{3}=c_{4}=0$. In this way, we apply the evolution equation on surfaces in each frequency and phase subspace, that is, on each $\mathrm {SE}(2)_{(\omega ,\phi )}$ manifold, which is the submanifold with elements $(q,\theta )$ representing the points $(q,\theta, \omega ,\phi )\in \mathcal{M}$ with fixed *ω* and *ϕ*. In this framework the metric $(g_{ij})$ boils down to

34 $$ (g_{ij})= \begin{pmatrix} 1+c_{1} (X_{1} u)^{2} & \sqrt{c_{1}c_{2}}X_{1}uX_{2}u \\ \sqrt{c_{1}c_{2}}X_{2}\mathit{uX}_{1} u & 1+c_{2} (X_{2} u)^{2} \end{pmatrix} . $$

We choose $c_{1}$ and $c_{2}$ suitably by regarding the fixed *ω* values.

There are two reasons for employing the reduced setting. First, we would like to avoid, by choosing $c_{3}=0$, excessive diffusion in the direction of the vector field $-\sin \theta \partial _{x} +\cos \theta \partial _{y}$, which is the first part of $X_{3}$. We already have sufficient diffusion in this direction due to the commutator $[X_{1}, X_{2}]$. Direct application of $X_{3}$ introduces excessive diffusion in orthogonal directions to the object boundaries, which is not desired since it may destroy object boundaries and contour structures in the input image. Moreover, the diffusion in phase results in multiplication of the evolving output responses by a constant since (see (1) and (9))

$$ \partial _{s} O^{I}(q,z)=v O^{I}(q,z), $$

where $v=1$ for all output responses (see [38]). Second, we eliminate the use of $X_{4}$ by fixing $c_{4}=0$ to reduce the computational load. We perform in this way a Laplace–Beltrami procedure in each frequency channel by avoiding any interaction between different frequency channels. It is an approximation of the exact flow. Yet, it captures the frequency content of the input image. This provides a diffusion where the dominant frequency components determine the resultant image in an analogous way to the multiscale left-invariant diffusion procedures presented in [36, 37, 47]. It is thanks to the lifting representing dominant frequency components with large output response values in $\mathcal{M}$. This reduced version results in multiple Laplace–Beltrami procedures applied in the three-dimensional sub-Riemannian geometry $\mathrm {SE}(2)_{(\omega, \phi )}$ at each frequency *ω* instead of the five-dimensional sub-Riemannian geometry $\mathcal{M}$. Finally, the vector fields $X_{3}$ and $X_{4}$ play a role rather in image inpainting (where the diffusion in $X_{1}$ and $X_{2}$ directions is not desired) than image enhancement.

Although in the present study we provide no results related to the image inpainting task of the Laplace–Beltrami procedure, we would like to mention a few related points. The use of the vector field $X_{3}$ becomes important in texture image inpainting. In that case, on the contrary to the enhancement, we would like to have information flow in orthogonal directions to the object boundaries and reduce the flow along the boundaries. In that case, since also the spatial frequency of the texture patterns have a great importance, we would like to keep the track of the frequency and phase of the evolving output responses. This requires fixing $c_{1}$ and $c_{2}$ to zero instead of $c_{3}$ and $c_{4}$ in that case.

### Implementation of the algorithm

#### The algorithm

We present the steps of our algorithm based on (33) by starting from the initial image function $I:\mathbb {R}^{2}\simeq M\rightarrow \mathbb {R}$ at $q\in M$.

1. Lift the image $I(q)$ to $O^{I}(q, p)$ by using (9). Choose this output as the initial value $u_{\vert t=0}$ of the solution to (33) at time $t=0$.
2. Denote the discrete step in time by Δ*t*. At the *k*th iteration (i.e., $t=k\Delta t$), compute the result of the discretized version *L̄* (of the operator *L*) applied on the current value of *u* at time instant *t* as $\bar{L} u(t)$ and update the solution and the value of $u(t)$ by using (33) as follows:
  $$ u(t+\Delta t)=u(t) +\Delta t \bar{L}u(t). $$
3. Repeat step 2 until the final time $T=(\text{number of iterations})\times \Delta t$ is achieved.
4. Apply the inverse Gabor transform given by (10) on $u(T)$.

#### Discrete simple cell output responses

We discretize the image function *I* on a uniform spatial grid as

35 $$ I[i,j]=I(i\Delta x, j\Delta y) $$

with $i,j\in \{1,2,\dots,N\}$ (*N* is the number of samples in spatial dimensions) and $\Delta x,\Delta y\in \mathbb {R}^{+}$ denoting the pixel width (in general, we use square images as input image, and we fix $\Delta x=\Delta y=1$ in terms of pixel unit). Furthermore, the discretized simple cell response $O^{I}(q_{1,i},q_{2,j},\theta _{k},\omega _{l},\phi _{m})$ of $I[i,j]$ on uniform orientation, frequency, and phase grids with points $\theta _{k}=k\Delta \theta $, $\omega _{l}=l\Delta \omega $, and $\phi _{m}=m\Delta s$ ($k\in \{1,2,\dots, K\}$, $l\in \{1,2,\dots,L\}$, $m\in \{1,2,\dots, M\}$ (where we denote the number of samples in the orientation dimension by *K*, in the frequency dimension by *L*, and in the phase dimension by *M*, and the distances between adjacent samples in the orientation dimension by Δ*θ*, in the frequency dimensions by Δ*ω*, and in the phase dimension by Δ*s*) is denoted by

36 $$ O^{I}[i,j,k,l,m]=O^{I}(q_{1,i},q_{2,j}, \theta _{k},\omega _{l},\phi _{m}), $$

where $q_{1,i}=i\Delta x$ and $q_{2,j}=j\Delta y$.

In this case the discrete version of the Gabor function given by (8) is written as

37 $$ \varPsi _{[i,j,k,l,m]}[\tilde{i},\tilde{j},\tilde{n}]=\varPsi _{(q_{1,i}, q_{2,j}, \theta _{k}, \omega _{l}, \phi _{m})}( \tilde{x}_{\tilde{i}}, \tilde{y}_{\tilde{j}},\tilde{s}_{\tilde{n}}), $$

where $\tilde{i},\tilde{j}\in \{1,2,\dots,\tilde{N}\}$, $\tilde{k}\in \{1,2,\dots,\tilde{K}\}$, $\tilde{n}\in \{1,2,\dots,\tilde{M}\}$. Then we fix $s_{\tilde{n}}=0$ (i.e., $\tilde{n}=0$) in the reduced framework (which was explained in Sect. 4.1.1) and write the discrete cell response obtained from the image $I[i,j]$ via the discrete Gabor transform as follows:

38 $$ O^{I}[i,j,k,l,m]=\sum_{\tilde{i},\tilde{j}}\varPsi _{[i,j,k,l,m]}[ \tilde{i},\tilde{j},0] I[\tilde{i},\tilde{j}]. $$

The time correspondence in the discrete case is represented by the time index $h_{p}$, where the time interval is discretized by $P\in \mathbb{N}^{+}$ samples, and $h_{p}$ represents the time instant $h_{p}=p\Delta t$ with Δ*t* satisfying $T=P\Delta t$ and $p\in \{1,2,\dots,P\}$. In this case the discretized Gabor coefficient is written as

39 $$ O^{I,h_{p}}[i,j,k,l,m]=O^{I,h_{p}}(q_{1,i},q_{2,j}, \theta _{k},\omega _{l}, \phi _{m})=u(p\Delta t). $$

#### Explicit scheme with finite differences

Here we provide the discrete scheme related to the numerical approximation of the algorithm. We propose an explicit finite difference scheme to iterate the evolution equation (33). The reason for choosing an explicit scheme is that an implicit scheme requires large memory in our four-dimensional (reduced) anisotropic framework.

We obtain the explicit scheme first by writing (33) in terms of the horizontal vector fields $X_{1}$, $X_{2}$, $X_{3}$, and $X_{4}$ given in (12). Then following Unser [102] and Franken [49], we implement the horizontal vector fields by using central finite differences that are interpolated by B-splines on a uniform spatial sample grid. Note that B-spline interpolation is required since not all horizontal vectors are aligned with the spatial sample grid.

The interpolation is achieved by determining the coefficients $b(i,j)$ in

40 $$ s(x,y)=\sum_{i,j\in Z}b(i,j)\rho (x-i,y-j) $$

in such a way that the spline polynomial $s(x,y)$ with the B-spline basis functions $\rho (x-i, y-j)$ coincides with the horizontal derivatives of the output $O^{I}$ at the grid points. For example, in the case of the first horizontal derivative $X_{1} O^{I}$, the condition $s(i\Delta x, j\Delta y)=X_{1}O^{I}[i,j,k,l,m]$ must hold if we consider a discrete output as explained in Sect. 4.2.2. We refer to the explanations of Unser [102] for details.

We fix $\Delta x=\Delta y=1$ and define

41 $$\begin{aligned} \begin{aligned} &e^{k}_{\xi }:= \bigl(\Delta x\cos (\theta _{k}),\Delta y\sin (\theta _{k})\bigr), \\ &e^{k}_{\eta }:= \bigl(-\Delta x\sin (\theta _{k}),\Delta y\cos (\theta _{k})\bigr). \end{aligned} \end{aligned}$$

**Figure 3:**
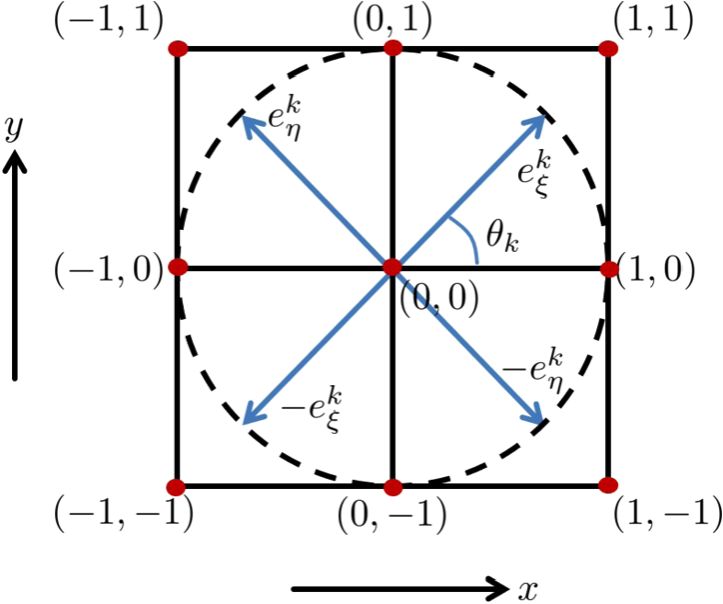
(Adapted from Franken [48]) Illustration of the vectors $e^{k}_{\xi }$ and $e_{\eta }^{k}$ at $(0,0)$ with $\Delta x=\Delta _{y}=1$

See Fig. 3 for an illustration of those vectors. We write the central finite differences of the first-order horizontal derivatives as

42 $$\begin{aligned} \begin{aligned} &X_{1} O^{I,h_{p}}[i,j,k,l,m]\approx \frac{1}{2\Delta x}\bigl(O^{I,h_{p}}\bigl(q+e^{k}_{ \xi }, \theta _{k},\omega _{l},\phi _{m}\bigr) -O^{I,h_{p}}\bigl(q-e^{k}_{\xi }, \theta _{k}, \omega _{l},\phi _{m}\bigr)\bigr), \\ &X_{2} O^{I,h_{p}}[i,j,k,l,m]\approx \frac{1}{2\Delta \theta } \bigl(O^{I,h_{p}}(q, \theta _{k+1},\omega _{l},\phi _{m}) -O^{I,h_{p}}(q,\theta _{k-1},\omega _{l}, \phi _{m})\bigr), \end{aligned} \end{aligned}$$

and of the second-order horizontal derivatives we use as

43 $$\begin{aligned} \begin{aligned} X_{1}X_{1}O^{I,h_{p}}[i,j,k,l,m] \approx{} &\frac{1}{(\Delta x)^{2}} \bigl(O^{I,h_{p}}\bigl(q+e^{k}_{\xi }, \theta _{k},\omega _{l},\phi _{m} \bigr)-2O^{I,h_{p}}(q, \theta _{k},\omega _{l},\phi _{m}) \\ &{} +O^{I,h_{p}}\bigl(q-e^{k}_{\xi },\theta _{k},\omega _{l},\phi _{m}\bigr) \bigr), \\ X_{2}X_{2} O^{I,h_{p}}[i,j,k,l,m]\approx{}& \frac{1}{(\Delta \theta )^{2}} \bigl(O^{I,h_{p}}(q,\theta _{k+1}, \omega _{l},\phi _{m})-2 O^{I,h_{p}}(q,\theta _{k},\omega _{l},\phi _{m}) \\ &{} +O^{I,h_{p}}(q,\theta _{k-1},\omega _{l},\phi _{m}) \bigr). \end{aligned} \end{aligned}$$

Then the numerical iteration (discretized from step 2 of the algorithm provided in Sect. 4.2.1) with a time step $\Delta t>0$ is written as follows:

44 $$ \begin{aligned} O^{I,h_{p+1}}[i,j,k,l,m]={} & O^{I,h_{p+1}}(q_{i,1},q_{j,2}, \theta _{k},\omega _{l},\phi _{m}) \\ = {}& O^{I,h_{p}}{(q_{i,1},q_{j,2},\theta _{k}, \omega _{l},\phi _{m})}+ \Delta t \bar{L} O^{I,h_{p}}(q_{i,1},q_{j,2}, \theta _{k},\omega _{l}, \phi _{m}), \end{aligned} $$

where *L̄* represents the discretized version of *L* given in (32) (with coefficients $c=\{c_{1}>0,c_{2}>0,c_{3}=0,c_{4}=0\}$) in terms of the central finite differences.

#### Stability analysis

We must consider two points for the stability of our finite discrete scheme:

1. Suitable choice of the time step size Δ*t*,
2. Preserving the space homogeneity during the Laplace–Beltrami evolution.

The stability analysis for the $\mathrm {SE}(2)$ case is explained in [36, 47, 49], and [39] based on Gershgorin theory. We adapt the analysis to our reduced framework and find the upper limit for the time step Δ*t* as

45 $$ \Delta t\leq \frac{4s_{\theta }^{2} \frac{c_{1}}{c_{2}}}{1+2\sqrt{2} s_{\theta }\sqrt{\frac{c_{1}}{c_{2}}}+3s_{\theta }^{2}\frac{c_{1}}{c_{2}}- \lvert 1-s_{\theta }^{2}\frac{c_{1}}{c_{2}} \rvert }, $$

where $s_{\theta }=\frac{2\pi }{K}$ is the sampling distance between adjacent orientation samples. In our experiments the worst case corresponds to $c_{1}/c_{2}=0.25$. In that case, $\Delta t=0.1\leq 0.87$, which is in accordance with the upper bound given in (45).

The second point is due to that we sample each dimension by using a different number of samples. To perform sub-Riemannian diffusion by regarding the sample unit coherency, we must choose the parameters $c_{1}$, $c_{2}$ of the operator *L* in such a way that the space homogeneity of $\mathcal{M}$ is preserved. For this reason, we choose $c_{1}=1$ and $c_{2}=\beta ^{2}$.

### Experiments

We first show the effects of the numbers of frequency and orientation samples. Then we present our simulation results together with the results presented in [64] and [47] for a comparison. The method in [64] lifts a two-dimensional image to a four-dimensional geometry, where the orientation and scale of the image is represented explicitly, and then it applies on the lifted image a multiscale Laplace–Beltrami procedure with a Riemannian metric. In [47] the two-dimensional image is lifted to the sub-Riemannian geometry of $\mathrm {SE}(2)$ via a so-called cake wavelet transform. In the lifted geometry the orientation corresponding to each point on the two-dimensional image is represented explicitly. Then a left-invariant coherence-enhancing and crossing-preserving diffusion (which is named CED-OS in [47]) is applied on the lifted image. It is a nonlinear adaptive diffusion procedure, which uses the local features curvature, deviation from horizontality, and orientation confidence.

#### Gabor transform

The delicate point related to the lifting and inversion process is that the Gabor functions $\varPsi _{(q,\theta, \omega ,\phi )}(x,y,s)$ must be sampled (in orientation *θ*, frequency *ω*, and phase *ϕ* dimensions) in such a way that they cover all the spectral domain (i.e., they must fulfill the Plancherel formula [85]).

**Figure 4:**
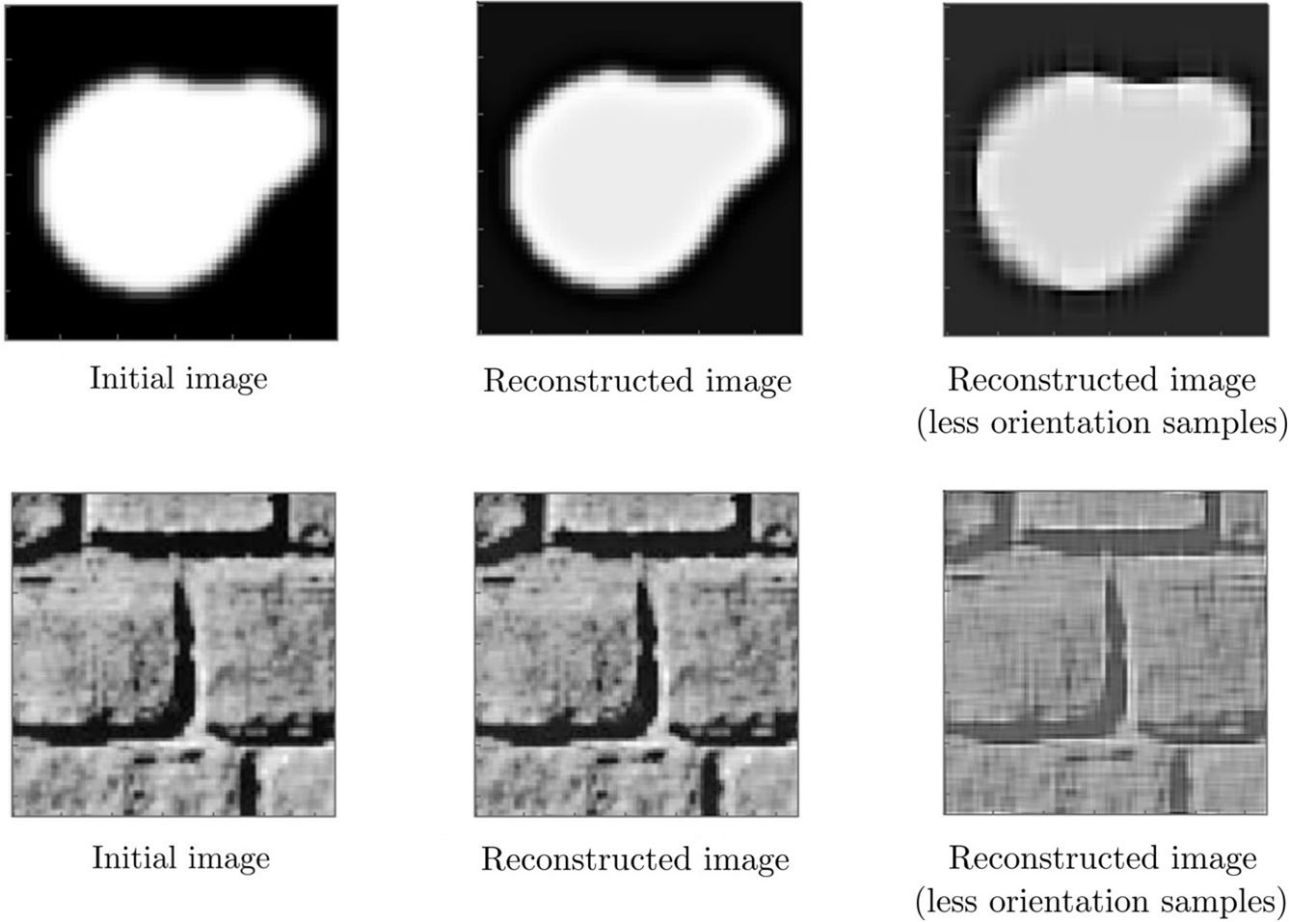
Examples of reconstructed images via transform and inverse transform procedure with Gabor functions, and the effect of number of orientation samples

**Figure 5:**
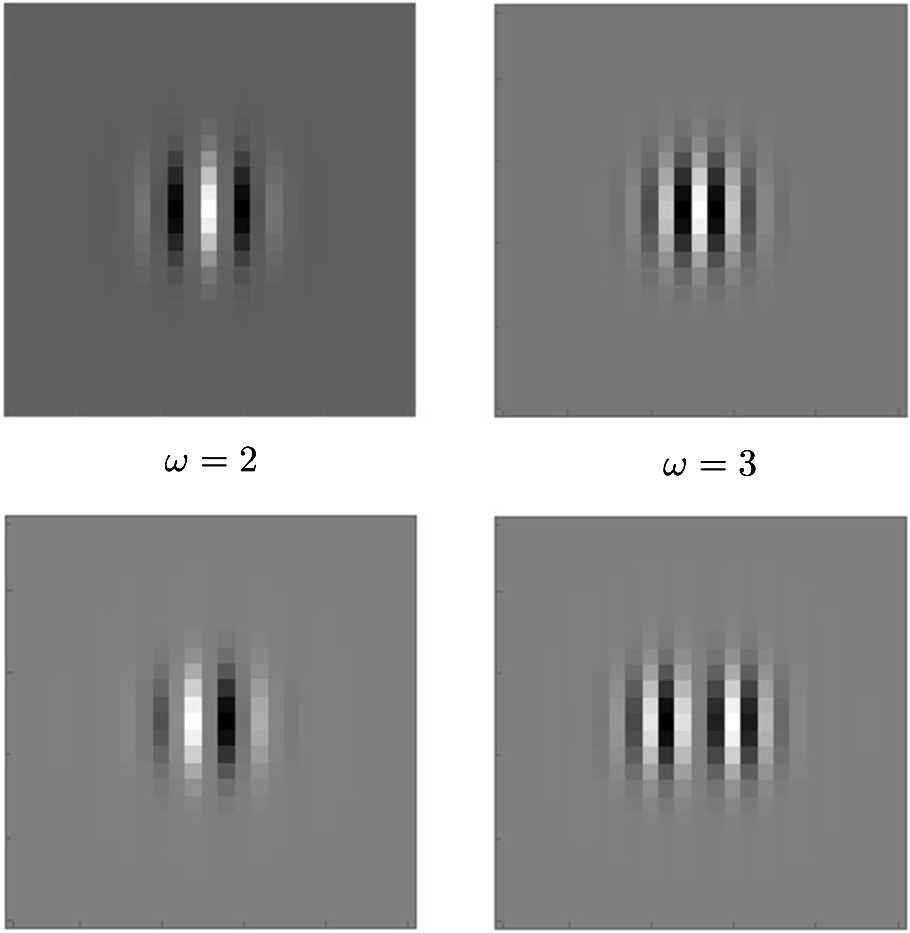
Examples of the Gabor filters used in the lifting procedure of Fig. 4. Top: Even parts of the Gabor functions with frequencies $\omega =2,3$. Bottom: Odd parts of the same Gabor functions

We present some results on the Gabor transform-inverse transform procedure associated with our setting and the effects of the number of samples in the orientation dimension in Fig. 4. We use the Gabor filter banks obtained from (6) and (8) with scale value of 2 pixels (the total filter size is 24 pixels) to lift the test images (see Fig. 5 for some examples of those Gabor functions). On the top row, we see the results related to an artificial $64\times 64$ test image (left), and at the bottom, we see the results related to a real $64\times 64$ test image (left) taken from Kimmel et al. [64] We see in the middle and right columns those two images now transformed and then inverse transformed with different numbers of orientation samples. We sample the space at frequencies $\omega \in \{0.25, 0.5,\dots, 1.25, 1.375,\dots, 2.25,2.3125,\dots, 3.25 \}$, orientations $\theta \in \{\frac{2\pi }{32},\frac{4\pi }{32},\dots,\frac{62\pi }{32} \}$ (middle) and $\theta \in \{0,\frac{2\pi }{8},\dots, \frac{14\pi }{8} \}$ (right), and phases $\phi \in \{0, \frac{\pi }{8},\dots, \frac{15\pi }{8}\}$. We observe that the decrease in the number of orientation samples reduces the quality of the transformation procedure noticeably in both test images.

**Figure 6:**
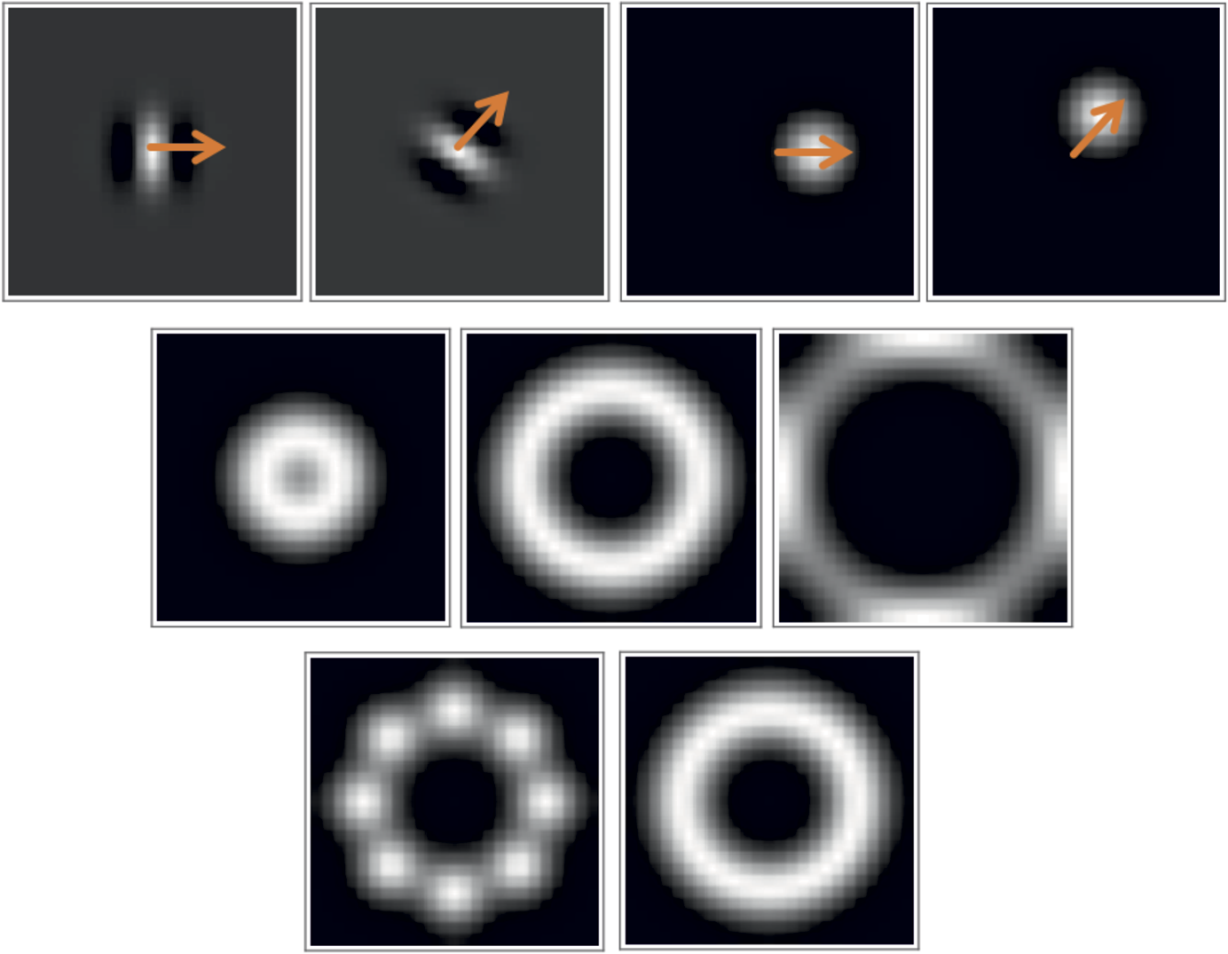
Top: Real part of a Gabor function (left) and its rotated version (middle right) together with their counterparts in the spectral domain in the same order (right and middle right). Orange arrows highlight the rotation angle. Middle: Set of rotated Gabor functions (in the spectral domain) corresponding to the frequency values $\omega =1,2,3$ in the same order from left to right. Bottom: The set of rotated Gabor functions in the spectral domain where the number of orientation samples are 8 and 16 rotation angles

Discrete orientation and frequency sampling of the Gabor functions used in the Gabor transform must be done in such a way that the Fourier transform of the filter bank must cover the whole spectral domain (due to the Parseval–Plancherel identity [81, 85]). This is essential for the reconstructability (see (10)) of the transformed signal. See Fig. 6 for the effect of discretization in orientation and frequency on the coverage of the Gabor transform in the spectral domain.

Discrete orientation and frequency sampling of the Gabor functions used in the Gabor transform must be done in such a way that the Fourier transform of the filter bank must cover the whole spectral domain (due to the Parseval–Plancherel identity [81, 85]). This is essential for the reconstructability (see (10)) of the transformed signal. See Fig. 6, where we show the effects of changing frequency and orientation of the Gabor functions and their effects on the spectral domain coverage. As the number of frequency and orientation samples decreases, the coverage becomes weaker, resulting in a degenerate reconstruction from the Gabor transform (see Figs. 4 and 11).

#### Enhancement

The lifting procedure is performed by the Gabor filters of the type given by (6) and (8) with $\text{scale}=2$ pixels (the filter size is $12\times \text{scale}=24$ pixels) and time step $\Delta t=0.1$ in the experiments.

**Figure 7:**
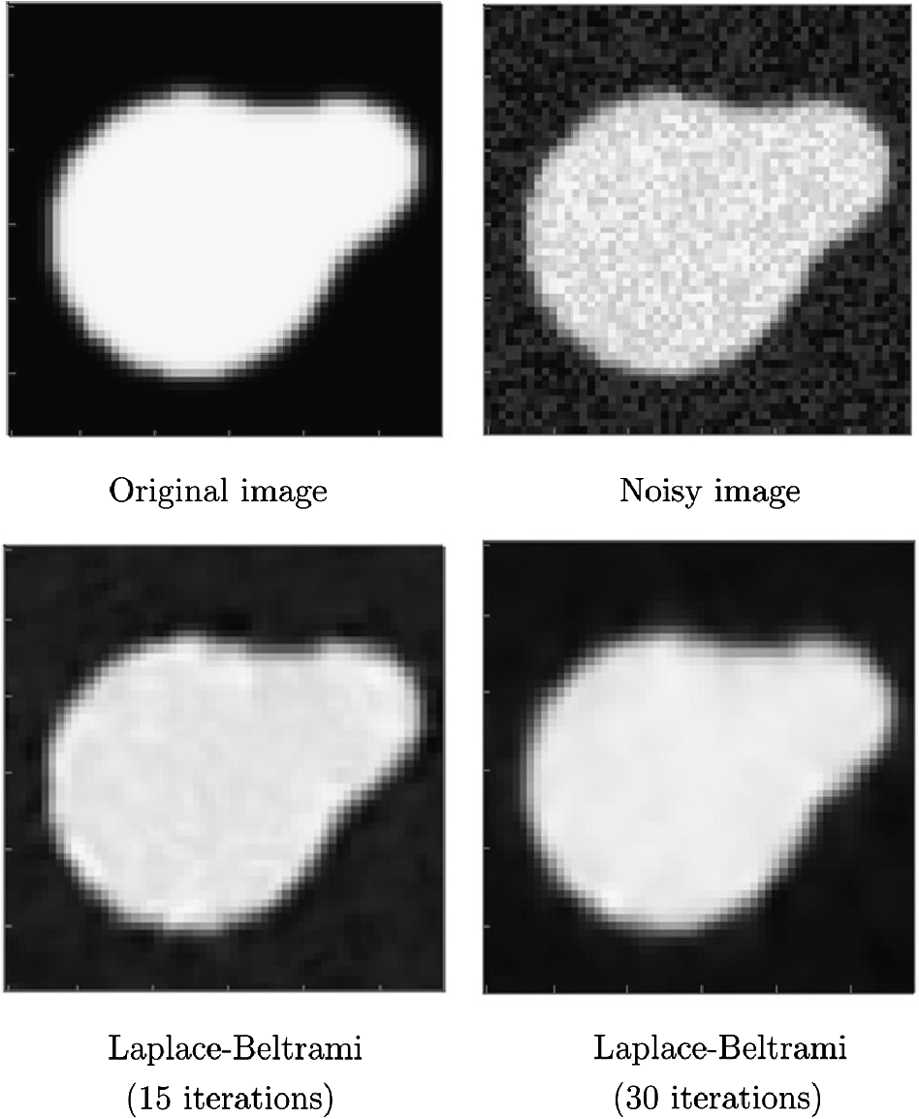
Top: The original $64\times 64$ image (left) and the noisy version (right). Bottom: The results of the Laplace–Beltrami procedure

In Fig. 7, we see the results of the enhancement procedure applied on an artificially produced $64\times 64$ grayscale test image with white noise. The lifting is achieved with frequency samples $\omega \in \{0.25, 0.5,\dots, 1,1.125,\dots, 2.25 \}$, phase samples $\phi =\{ 0,\frac{\pi }{8},\dots,\frac{\pi }{2}\}$, and orientation samples $\theta \in \{0, \frac{2\pi }{16},\frac{4\pi }{16},\dots, \frac{30\pi }{16}\}$. Note that $\text{number of orientations}=16$, and thus $\beta =\frac{\text{number of orientations}}{\text{image size}}=0.25$. To fulfill physical unit coherency, we choose $c_{1}=1$ and $c_{2}=\beta ^{2}$. The experiments are done with 15 and 30 iterations.

**Figure 8:**
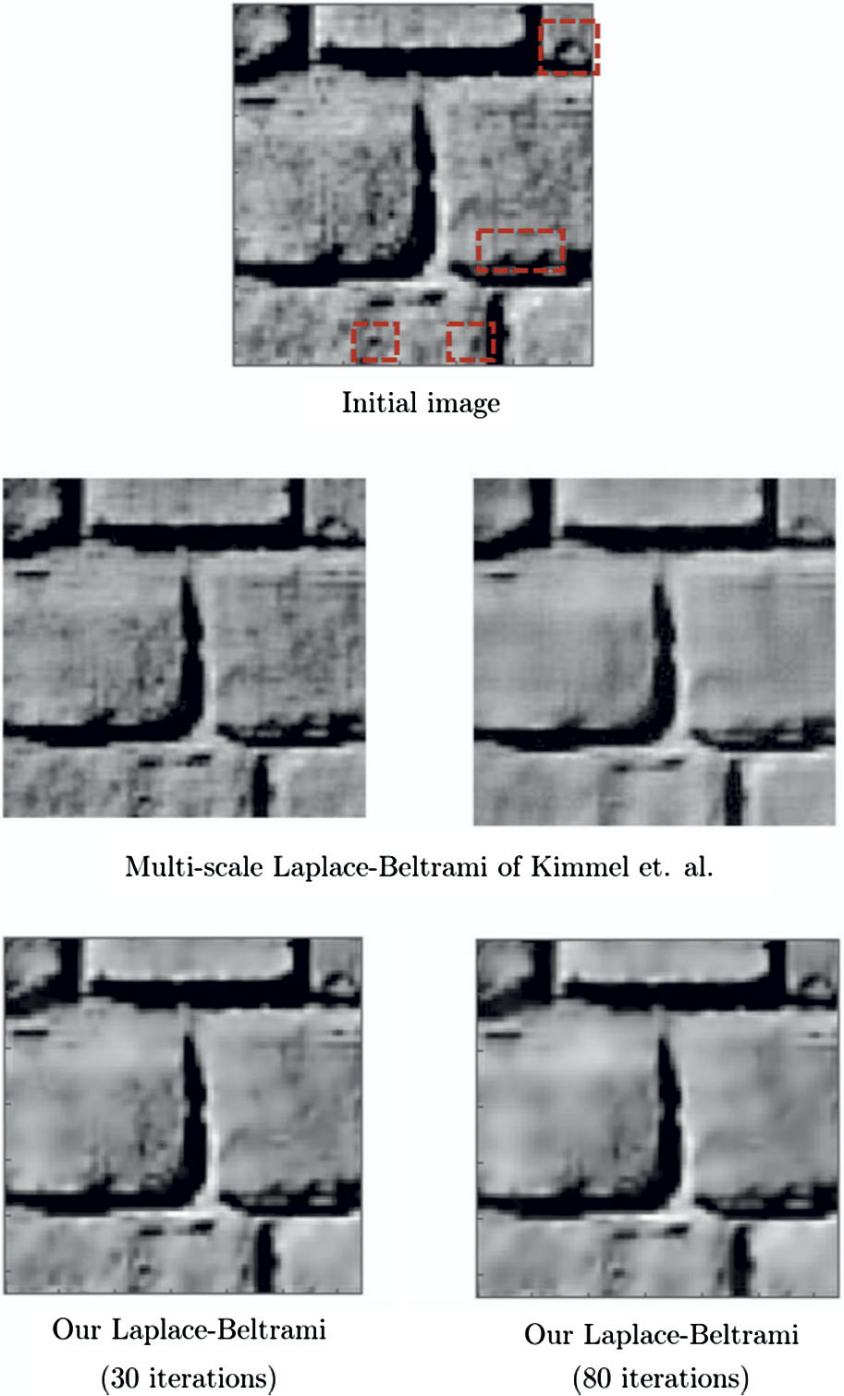
Top: The initial image taken from [64]. Middle: The results obtained by Kimmel et al. [64]. Bottom: The results of our Laplace–Beltrami procedure

We continue with Fig. 8, where we apply our procedure on a real $128\times 128$ image taken from Kimmel et al. [64], who use a multiscale Laplace–Beltrami procedure with fixed frequency. We use the same phase and orientation samples as in the case of Fig. 7, but we employ the frequency samples $\omega \in \{ 0.25, 0.5,\dots, 1,1.125,\dots, 2.25, 2.3125,\dots, 3 \}$ for the lifting. Here the coefficients $c_{1}$, $c_{2}$ are chosen as in the case of Fig. 7. We perform the experiments with 30 and 50 iterations.

**Figure 9:**
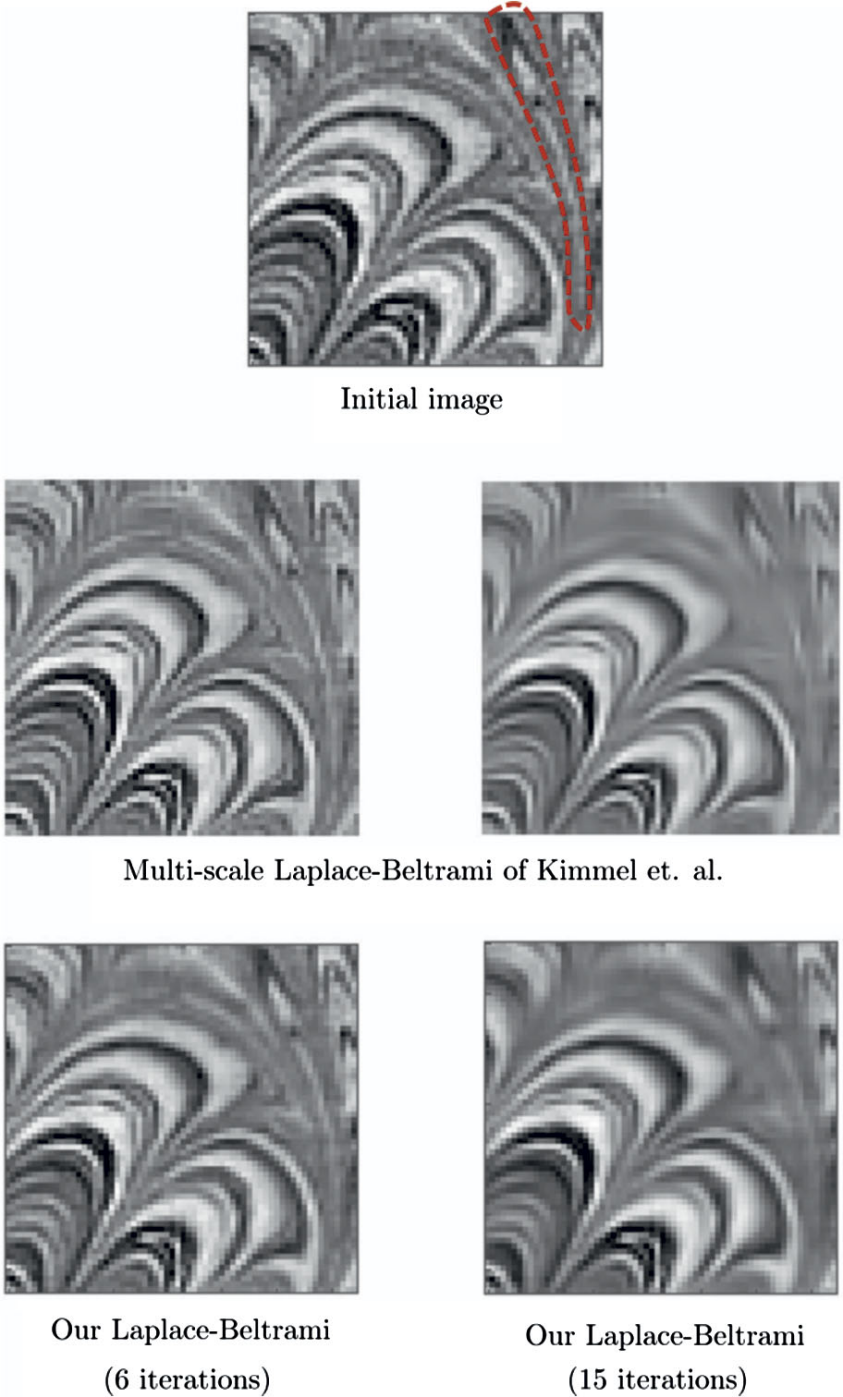
Top: The initial image taken from [65]. Middle: The results obtained by Kimmel et al. [65]. Bottom: The results of our Laplace–Beltrami procedure

In Fig. 9, we show the results related to our Laplace–Beltrami procedure applied on another real image, with dimensions $64\times 64$, taken from Kimmel et al. [64]. We use the same sampling parameters as in the previous case of Fig. 8 for the lifting. We perform our Laplace–Beltrami procedure with 6 and 15 iterations. The results are presented together with the multiscale Laplace–Beltrami results obtained by Kimmel et al. [64] for a qualitative comparison. In Figs. 8 and 9, we highlight with a red dashed curve a few details related to contextual structures, particularly related to the frequency feature: Our algorithm (bottom right) takes advantage of different frequencies present in the images and therefore can preserve texture structures and preserves these structures better than that of Kimmel et al. [64] (middle right).

**Figure 10:**
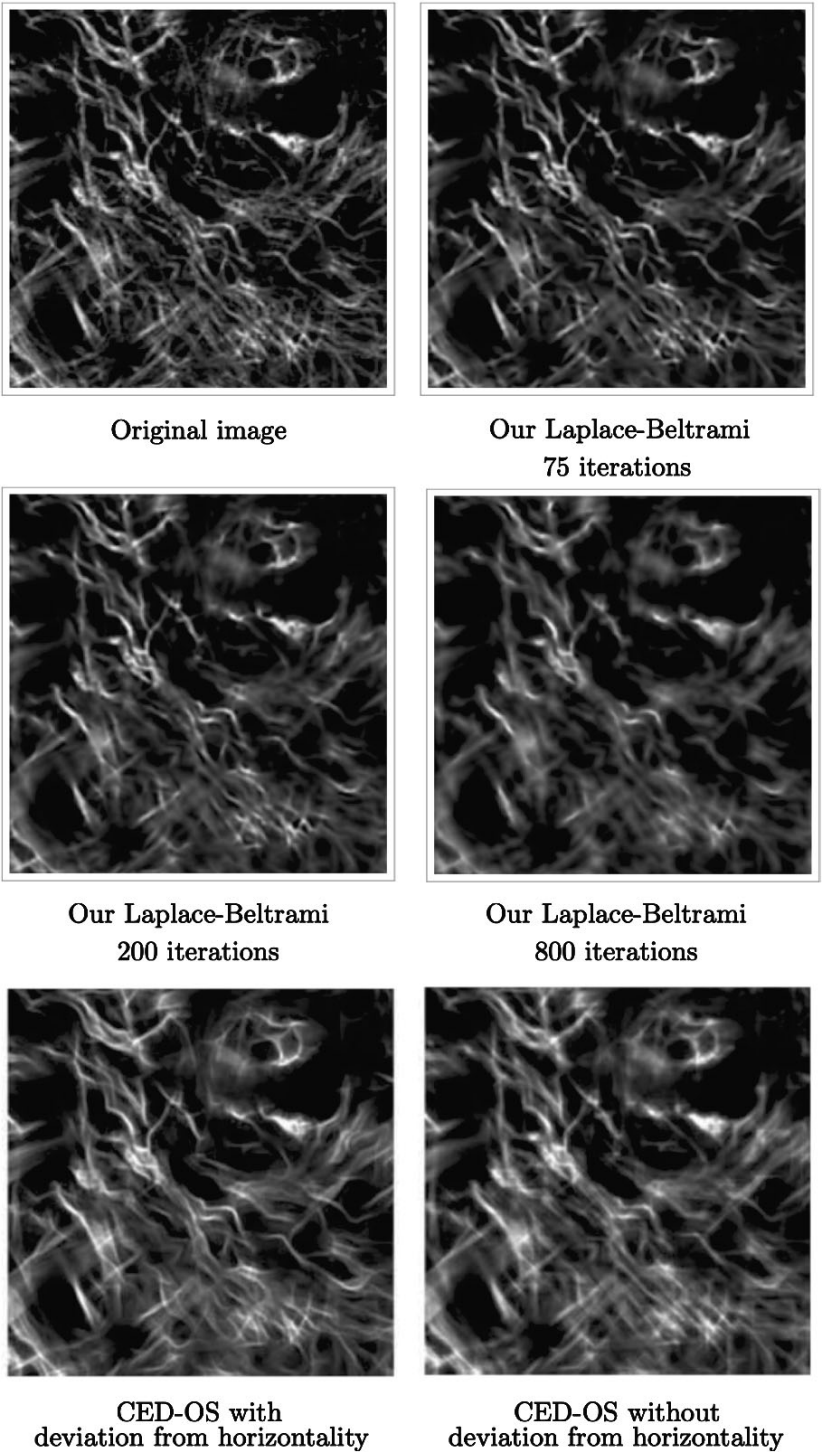
Top: The original image taken from [47] (left); the results obtained by our method via 75 and 200 iterations (middle and right, respectively). Bottom: The results of CED-OS taken from [47]. The result with deviation from horizontality and without deviation from horizontality are given on the left and right, respectively

We compare our technique to the enhancement method provided in [47], where nonlinear adaptivity mechanisms are combined with a left-invariant diffusion procedure performing in the sub-Riemannian geometry of $\mathrm {SE}(2)$. In Fig. 10, we provide the results of our algorithm applied with different numbers of iterations on a $256\times 256$ grayscale image taken from [47]. We use the Gabor filter banks obtained from (6) and (8) with scale value of 2 pixels (total filter size is 48 pixels) to lift the test images. We sample the space at frequencies $\omega \in \{1.45, 1.51, 1.58, 1.66, 1.74, 1.82, 1.91, 2, 2.09, 2.19\}$ and orientations $\theta \in \{\frac{2\pi }{16},\frac{4\pi }{16},\dots,\frac{32\pi }{16} \}$. We use $c_{1}=1$ and $c_{2}=\beta ^{2}$ as before and choose the time step $\Delta t=0.1$. Here we note that CED-OS algorithm presented in [47] performs an adaptive diffusion in the three-dimensional sub-Riemannian geometry where only the orientation feature is represented explicitly. Despite the fact that it does not perform a multiscale or multifrequency wavelet transform, it provides good enhancement results thanks to the nonlinear adaptation mechanisms taking into account the local features curvature, deviation from horizontality, and orientation confidence. Our results are comparable to those of CED-OS when such nonlinear adaptive mechanisms are not used (see the bottom left and bottom right in comparison to the middle left of Fig. 10). At this point, we remark that the reduced model framework is flexible in the sense that it contains already $\mathrm {SE}(2)$ at each frequency separately. Hence $\mathrm {SE}(2)$ can be considered as a particular case of our model framework. There is no obstacle to employing nonlinear operators in this setting (as long as they are adequately used in $\mathrm {SE}(2)$) as in the case of CED-OS algorithm explained in [47] or as in the case of diffusion-concentration procedure presented in [25]. We can choose the desired frequency channel in our model framework and apply such nonlinear algorithms in the corresponding $\mathrm {SE}(2)$ to that frequency channel.

**Figure 11:**
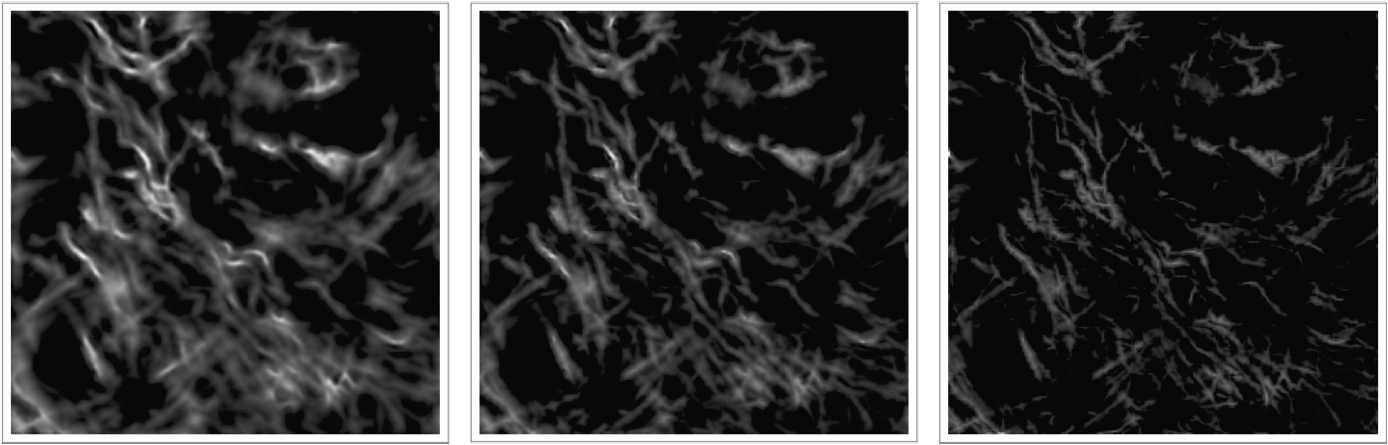
Left: The result of our multifrequency Laplace–Beltrami procedure applied on an image taken from [47] (see also Fig. 10). Middle: The result of the single-frequency Laplace–Beltrami procedure with $\omega =1.91$. Right: The result of the single frequency Laplace–Beltrami procedure with $\omega =2.19$

Finally, we see in Fig. 11 a comparison of our multifrequency procedure to its single-frequency counterpart. The procedure and simulation parameters are the same as given in the case of Fig. 10. We observe that the single-frequency Laplace–Beltrami procedure results in a lower quality enhancement in comparison to the multifrequency Laplace–Beltrami procedure.

## Conclusion

In this paper, we have shown that the multifeature selective simple cells and the associated V1 functional geometry can be modeled starting from a suitably chosen receptive profile, which in our framewor kwas the extended Gabor function. We have derived the whole model sub-Riemannian geometry and the corresponding horizontal connectivity directly from the receptive profile. In addition to this construction of the model, we have also provided an image processing application employing our model framework, image enhancement via a sub-Riemannian Laplace–Beltrami procedure. We have provided the algorithm and its discretization explicitly as well as some experimental results. We have also mentioned that, in fact, the enhancement procedure could be switched to an image inpainting procedure via a modification of the reduced metric used for the enhancement.

As far as the complexity and the richness of the visual semantics are considered, it is natural to think that the visual system samples all features once it is given a visual input to find a unique correspondence between the visual input and output. The necessity of handling such a variety of images results in a suitable compromise in rendering the visual features. This compromise manifests itself as the visual system being restricted to a psychophysically and neurophysiologically relevant architecture. As such, the visual system is limited in highly specialized tasks, which are used in several domains such as medical image analysis, image processing, radar imaging, and computer and robotic vision.

Our model is not particularly for image processing. It is not motivated by image processing problems and it should not be interpreted as an image processing model. Firstly, image processing models are specialized on specific visual tasks, which are required generally for a certain category of images (medical images, radar images, etc.). They are not necessarily restricted to psychophysical and neurophysiological findings providing information about the architecture of the visual system. Secondly, they can make use of several nonlinear mechanisms, which need not be motivated by any biological reasoning. Our model is motivated biologically, and it relies on the psychophysically and neurophysiologically relevant cortical architecture. It is a phenomenological model, and it provides a geometrical explanation for the cortical architecture, which is compatible with the architecture. Finally, the application of the model to image enhancement was provided to show effects of the use of spatial frequency by comparing it qualitatively to some other image enhancement algorithms.

## Footnotes

1 Spatial frequency refers to $\omega =\frac{2\pi }{\lambda }$ with wavelength $\lambda >0$ in our terminology.

## Abbreviations

V1: Primary visual cortex
MRI: Magnetic resonance imaging
